# Identification and panoramic analysis of drug response-related genes in triple negative breast cancer using as an example NVP-BEZ235

**DOI:** 10.1038/s41598-023-32757-4

**Published:** 2023-04-12

**Authors:** Jia Feng, Luchang Wang, Kaijiong Zhang, Sujiao Ni, Baolin Li, Jinbo Liu, Dongsheng Wang

**Affiliations:** 1 Department of Clinical Laboratory, The Affiliated Hospital of Southwest Medical University, Luzhou, Sichuan China; 2 Department of Clinical Laboratory, Sichuan Clinical Research Center for Cancer, Sichuan Cancer Hospital & Institute, Sichuan Cancer Center, Affiliated Cancer Hospital of University of Electronic Science and Technology of China, Chengdu, Sichuan China

**Keywords:** Breast cancer, Cancer microenvironment, Cancer models, Cancer therapy, Tumour biomarkers

## Abstract

Taking NVP-BEZ235 (BEZ235) as an example to screen drug response-related genes (DRRGs) and explore their potential value in triple-negative breast cancer (TNBC). Through high-throughput technique, multidimensional transcriptome expression data (mRNA, miRNA and lncRNA) of BEZ235-treated and -untreated MDA-MB-468 cell lines were obtained. Combined with transcriptome data of the MDA-MB-468 cells and TCGA-TNBC tissues, differential gene expression analysis and WGCNA were performed to identify DRRGs associated with tumor trait by simulating the drug response microenvironment (DRM) of BEZ235-treated patients. Based on DRRGs, we constructed a ceRNA network and verified the expression levels of three key molecules by RT-qPCR, which not only demonstrated the successful construction of a BEZ235-treated cell line model but also explained the antitumor mechanism of BEZ235. Four molecular subtypes related to the DRM with survival difference were proposed using cluster analysis, namely glycolysis subtype, proliferation depression subtype, immune-suppressed subtype, and immune-activated subtype. A novel prognostic signature consisting of four DRRGs was established by Lasso–Cox analysis, which exhibited outstanding performance in predicting overall survival compared with several excellent reported signatures. The high- and low-risk groups were characterized by enrichment of metabolism-related pathways and immune-related pathways, respectively. Of note, the low-risk group had a better response to immune checkpoint blockade. Besides, pRRophetic analysis found that patients in the low-risk group were more sensitive to methotrexate and cisplation, whereas more resistant to BEZ235, docetaxel and paclitaxel. In conclusion, the DRRGs exemplified by BEZ235 are potential biomarkers for TNBC molecular typing, prognosis prediction and targeted therapy. The novel DRRGs-guided strategy for predicting the subtype, survival and therapy efficacy, might be also applied to more cancers and drugs other than TNBC and BEZ235.

## Introduction

Triple-negative breast cancer (TNBC), accounting for 15–20% of all breast cancer (BC) cases, is one of the most aggressive BC subtypes^1^. TNBC treatments using chemotherapy and radiotherapy show limited therapeutic benefits, with the majority of patients still at high risk of relapse and development of distant metastasis^2^. TNBC exhibits poor prognosis compared with other BC subtypes due to their aggressive clinical features and lack of specific molecular targets. Therefore, the search for novel therapeutic and prognostic biomarkers will help to develop more effective TNBC treatment strategies.

Molecularly targeted therapy, which aims at mutations or dysregulated pathways leading to oncogenesis, is a popular modality of pharmacotherapy for cancer in recent years^3^. The PI3K/AKT/mTOR signaling pathway plays a crucial role in controlling cellular functions, including cell proliferation, metabolism, and survival. More and more studies have indicated that PI3K/AKT/mTOR pathway plays a critical role in TNBC^4–6^. NVP-BEZ235 (BEZ235), a dual PI3K/mTOR inhibitor that selectively inhibits class I PI3K (p110α, -β, -δ and -γ), mTORC1 and mTORC2 by reversibly binding to the ATP-binding sites of kinases and inhibiting their catalytic activity and signaling, has great potential as an antitumor drug. BEZ235 has antitumor activity (both in monotherapy and in combination with other anticancer agents) in multiple preclinical cancer models including BC, prostate cancer, sarcomas, non-small cell lung cancer, colorectal cancer and ovarian cancer^7–12^, which is currently undergoing evaluation in Phase I/II clinical trials for the treatment of tumors^13,14^. However, Research indicated BEZ235 inhibited PI3K signaling transiently and its therapeutic effects in BC were not efficient^15^. Therefore, the in-depth study of BEZ235 treatment and resistance-related mechanisms in TNBC is very necessary.

Measurements of the genetic characteristics and therapeutic responses of cell lines are well suited for the development of strategies to identify the most predictive molecular markers. Several researchers have made efforts to characterize relationships between genomic profiles and drug responses^16,17^. Drug response-related genes (DRRGs) can provide insights into drug antitumor or resistance mechanisms, thus facilitating drug development and personalized therapy. Du et al. identified 159 genes potentially correlated with the cytoxan response of BC patients, which had prognostic value^18^. Shen et al. identified 32 genes associated with multi drug response in ER- BC cell lines, and functional analysis showed that these genes are mainly enriched in signaling pathways related to metabolic function, and cell cycle processes affect drug response of cells^19^. However, studies on drug response genes-based biomarkers or signatures are limited in TNBC.

In our previously published study, BEZ235 was found to exert anti-tumor effects on TNBC cells (MDA-MB-468 and MDA-MB-231) by inhibiting Akt/mTOR pathway activity and stimulating mutant p53 degradation^20^. On this basis, we boldly combined the multi-dimensional transcriptome data of cell lines (BEZ235-treated and -untreated MDA-MB-468 cells) with tissues (Tumor vs. Normal) for the first time to identify BEZ235 response-related genes associated with tumor trait by simulating the DRM of TNBC patients treated with BEZ235. Based on these genes, we constructed a drug response-related ceRNA network, discovered four molecular subtypes related to the drug response microenvironment (DRM), and constructed an excellent four-gene prognostic risk prediction model. To elucidate the importance of DRRGs from the aspects of expression, function, regulatory mechanism, typing and prognostic value. These findings will help us to further dissect the anti-tumor effects and potential resistance mechanisms of BEZ235, and to provide a reliable experimental basis for the study of TNBC.

## Results

### Screening for BEZ235 response-related genes associated with tumor trait

**Figure 1:**
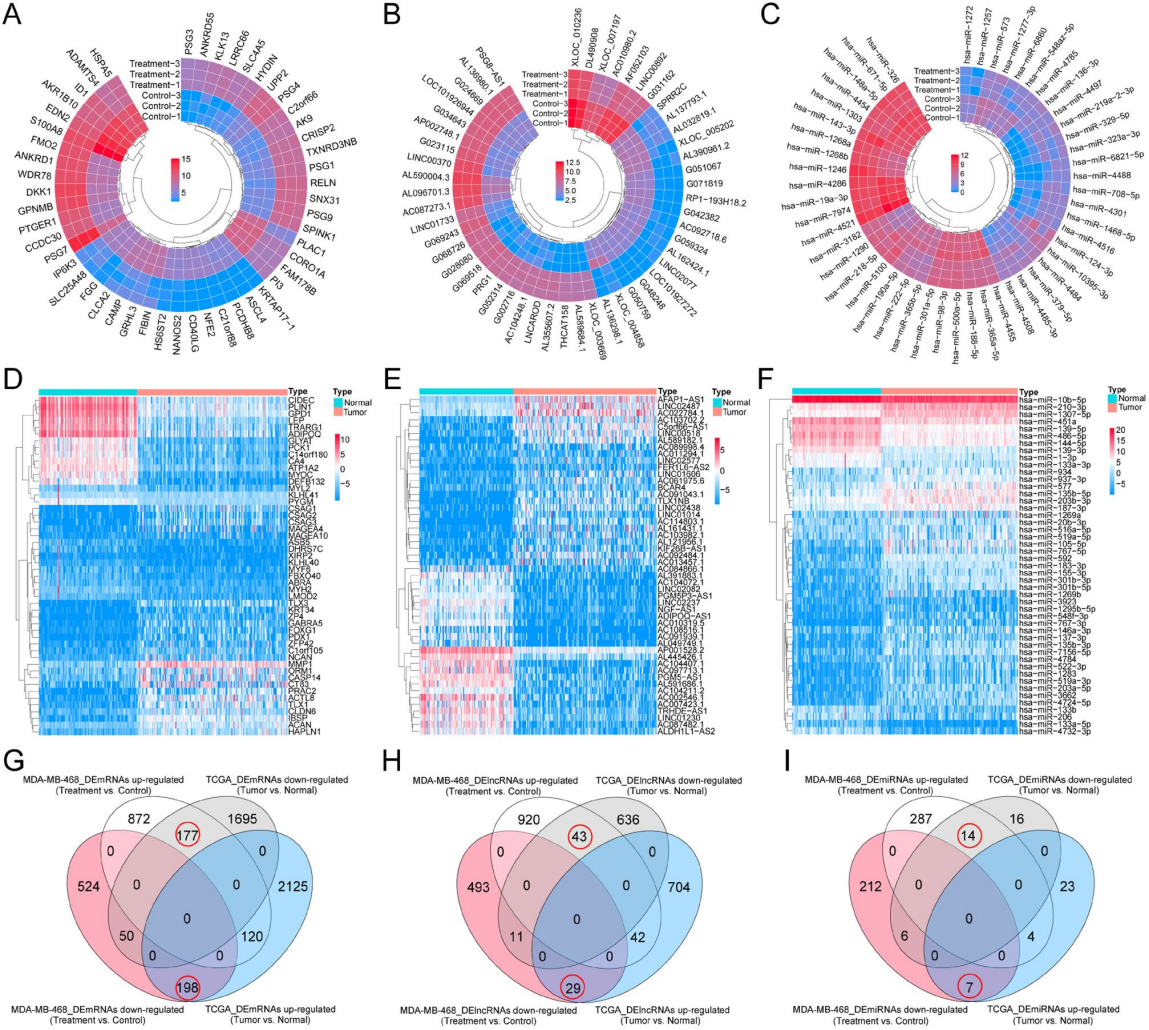
Screening for the BEZ235 response-related genes. (**A**–**C**) The heatmap showing the top 50 DEmRNAs, DElncRNAs, and DEmiRNAs between BEZ235-treated and control group, respectively. (**D**–**F**) The heatmap showing the top 50 DEmRNAs, DElncRNAs, and DEmiRNAs between tumor and normal group, respectively. (**G**–**I**) The Venn diagram was utilized to obtain BEZ235 response-related DElncRNAs, DEmRNAs and DEmiRNAs, respectively. DEmRNAs/DElncRNAs/DEmiRNAs: differentially expressed mRNAs/lncRNAs/miRNAs.

The flow chart of this study is shown in Fig. S1. Following the differential gene analysis, 1941 Differentially expressed mRNAs (DEmRNAs), l538 DElncRNAs, and 70 DEmiRNAs were obtained from MDA-MB-468 data set (Treatment vs. Control), and 4365 DEmRNAs, 1465 DElncRNAs and 530 DEmiRNAs from TCGA-TNBC data set (Tumor vs. Normal). The heatmaps depicted the expression levels of the top 50 DERNAs (Fig. 1A–F). Furthermore, Celligner web tool^21^ analysis found that MDA-MB-468 exhibited high TNBC transcriptome fidelity (Fig. S2). Therefore, the DERNAs obtained from the MDA-MB-468 dataset and the TCGA-TNBC dataset were crossed to obtain BEZ235 response-related genes that were up-/down-regulated in TNBC tissues but down/up-regulated in BEZ235-treated cell lines. Venn diagrams showed that 375 DEmRNAs, 72 DElncRNAs and 21 DEmiRNAs intersected between the two datasets (Fig. 1G–I).

**Figure 2:**
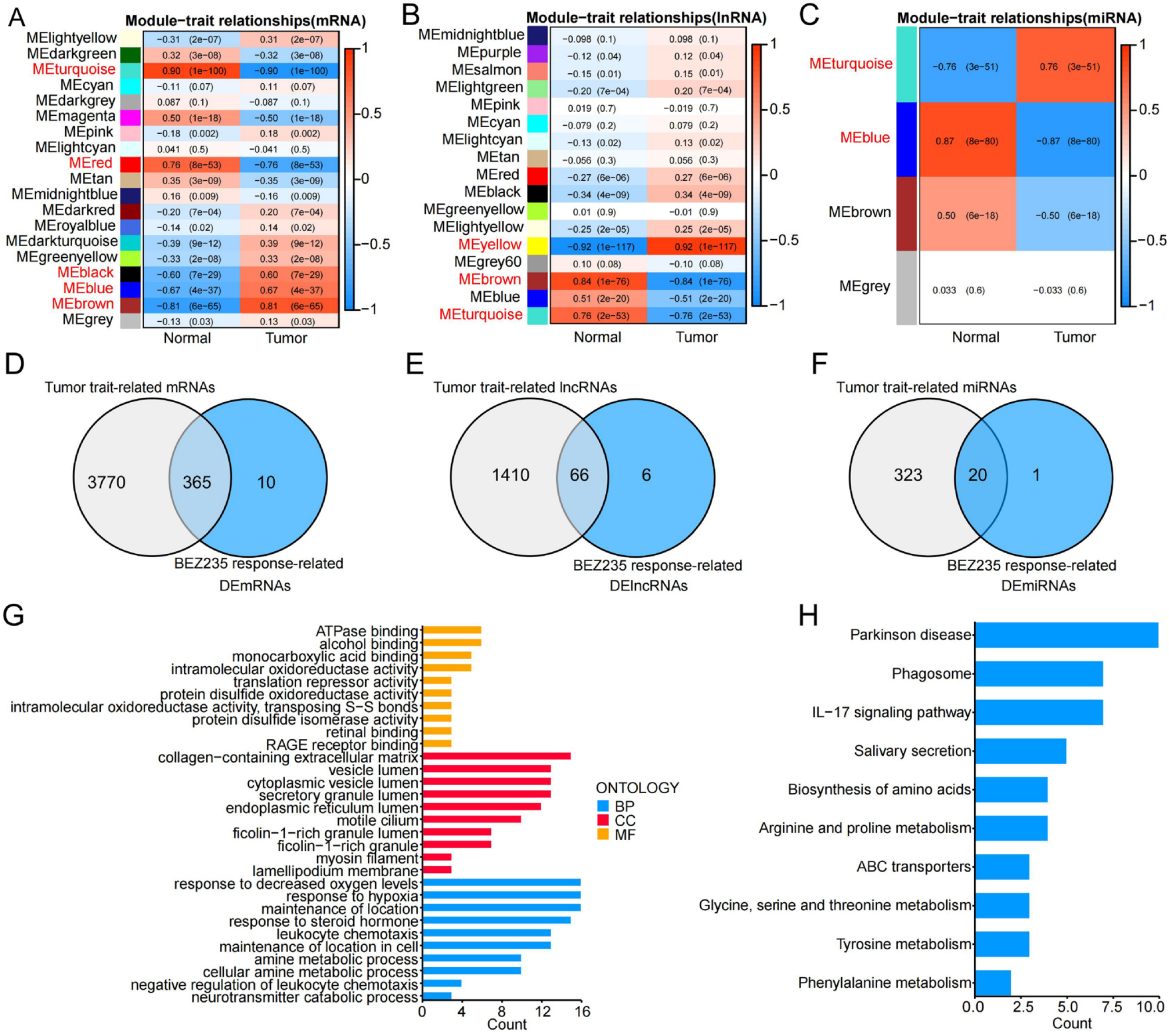
Screening for the BEZ235 response-related genes associated with tumor trait and function analysis. (**A**–**C**) The correlation of the modules with the tumor traits was shown in the heatmap. Each cell contained the corresponding correlation and *P* value. The red font represents the selected modules. (**D**–**F**) The Venn diagram was utilized to obtain tumor-related and BEZ235 response-related DERNAs. (**G**–**H**) Enrichment of GO and KEGG terms for BEZ235 response-related genes associated tumor trait. DERNAs: differentially expressed RNAs.

Soon afterwards, the mRNA, lncRNA and miRNA expression matrix were analyzed respectively based on the WGCNA algorithm in the TCGA-TNBC dataset, and 10 modules related to TNBC tumor trait were screened out, including 5 mRNA co-expression modules, 3 lncRNA co-expression modules, and 2 miRNA co-expression modules (Fig. 2A–C). The genes of the co-expression modules were intersected with BEZ235 response-related genes, and a total of 451 genes were obtained, including 365 DEmRNAs, 66 DElncRNAs, and 20 DEmiRNAs (Fig. 2D–F, Table S1). These results indicated that most of the BEZ235 response-related genes were associated with tumor trait.

Whereafter, we performed GO and KEGG analyses. Multiple GO terms, including hypoxia response, and KEGG pathways, including IL-17 signaling pathway, were revealed. The top 10 remarkable GO terms and KEGG pathways were shown in Fig. 2G–H, implying that these biological processes and pathways may be correlated with the BEZ235 response of TNBC patients.

### BEZ235 response-related ceRNA network was constructed to search for potential therapeutic targets

**Figure 3:**
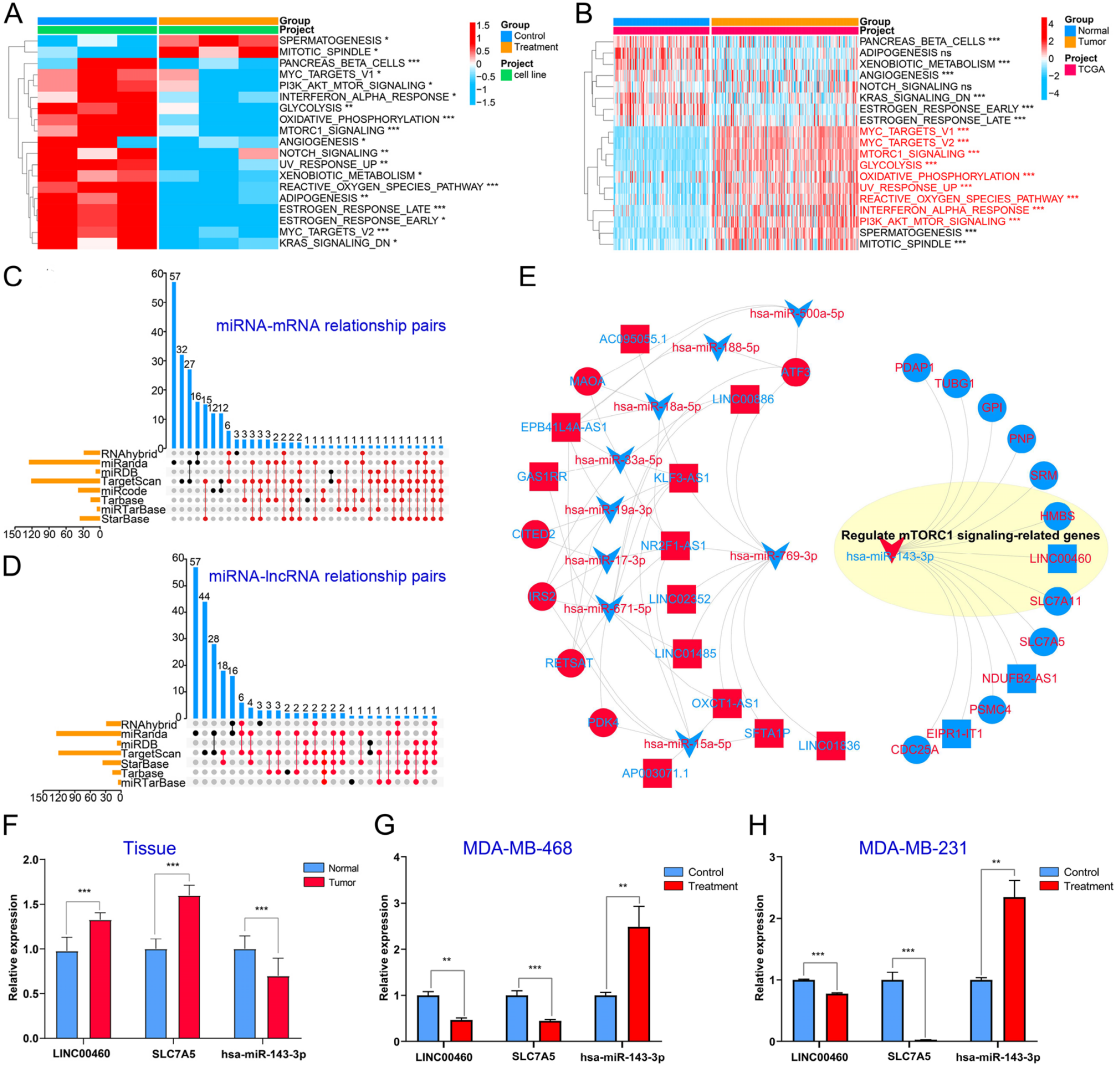
Construction of BEZ235-related ceRNA network. (**A**,**B**) GSVA analysis screened for differential Hallmark pathways between tumor and normal, and between BEZ235-treated and control group, respectively. The upset diagrams were used to screen the intersection miRNA-mRNA pairs (**C**) and miRNA-lncRNA pairs (**D**), and the dot-lines in red represented the selected relationship pairs. (**E**) BEZ235-related ceRNA network was built by Cytoscape. The red shape/font represents the genes upregulated in BEZ235-treated cell lines/TNBC tissue, and the blue shape is the opposite. (**F**–**H**) The RT-qPCR analysis was used to verify the expression levels of 3 genes, including LINC00460, SLC7A5and hsa-miR-143-3p. “***”*P* < 0.001, “**”*P* < 0.01, “*”*P* < 0.05.

Through GSVA analysis, nine markedly upregulated hallmark pathways in TCGA-TNBC tissues were found to be inhibited in BEZ235-treated MDA-MB-468 cell, including PI3K/AKT/mTOR signaling pathway and mTORC1 signaling pathway (logFC > 0.25, *P*_*FDR*_ < 0.05) (Fig. 3A,B). The finding implied that BEZ235 did inhibit the PI3K-/mTOR-related pathways, further confirming that drug-treated cell model was constructed successfully. Tumor cells can affect pathway activity by changing the expression of key genes thereby affecting the biological behavior of tumor cells. Hence, we tried to reveal the anti-tumor mechanism of BEZ235 from the perspective of ceRNA regulatory network. Subsequently, 365 BEZ235 response-related DEmRNAs associated with tumor traits were crossed with the related genes on these nine differential hallmark pathways to obtain 46 overlapping DEmRNAs (Table S2). The latter participated in the construction of ceRNA network as a candidate.

After the above analysis, 46 DEmRNAs, 66 DElncRNAs and 20 DEmiRNAs were available for the construction of the ceRNA network. Through a range of miRNA target prediction softwares and databases, we obtained miRNA-mRNA relationship pairs and miRNA-lncRNA relationship pairs, and displayed them by Upset diagrams (Fig. 3C,D). After strict construction criteria, a BEZ235 response-related ceRNA network was finally established in MDA-MB-468 cells by Cytoscape, which consisted of a sub-network centered on upregulated miRNAs and a sub-network centered on down-regulated miRNAs (Fig. 3E). In total, there were 67 relation pairs in this ceRNA network including 15 lncRNAs, 16 mRNAs, and 10 miRNAs (Table S3). We found that the down-regulated DEmRNAs except SRM all came from the mTORC1 signaling pathway and targeted by hsa-miR-143-3p, suggesting that hsa-miR-143-3p might be an important target for TNBC therapy^22^. Meanwhile, our data confirmed for the first time that BEZ235 mainly acted on mTORC1 signaling pathway from the perspective of ceRNA regulatory network, which was consistent with the main role of BEZ235^23^. Furthermore, a large number of genes in the sub-network centered on down-regulated DEmiRNAs were found to be associated with drug resistance or sensitivity, such as *ISR2*^24^, *NR2F1-AS1*^25^ and *hsa-miR-671-5p*^26^ implying these sub-network genes may be new indicators for TNBC treatment.

Moreover, we randomly selected genes from the ceRNA network to confirm their expression levels by RT-qPCR analysis. As shown in the Fig. 3F–H, the expression levels of LINC00460 and SLC7A5 mRNA were elevated in TNBC tissues and down-regulated in BEZ235-treated cells, while has-miR-143-3p was opposite. These suggest that LINC00460-has-miR-143-3p-SLC7A5 network may be involved in TNBC progression or BEZ235 therapy mechanism. Besides, we found that the expression trends of OXCT1-AS1, ATF3 were consistent with our analysis. While, there was no statistical difference in the expression level of hsa-miR-671-5p in tissues andMDA-MB-231 cells (Fig. S3). The above analysis confirms that our prediction has a certain reliability, but further experimental verification is still needed.

### There are four drug response-related subtypes with survival difference in TNBC

**Figure 4:**
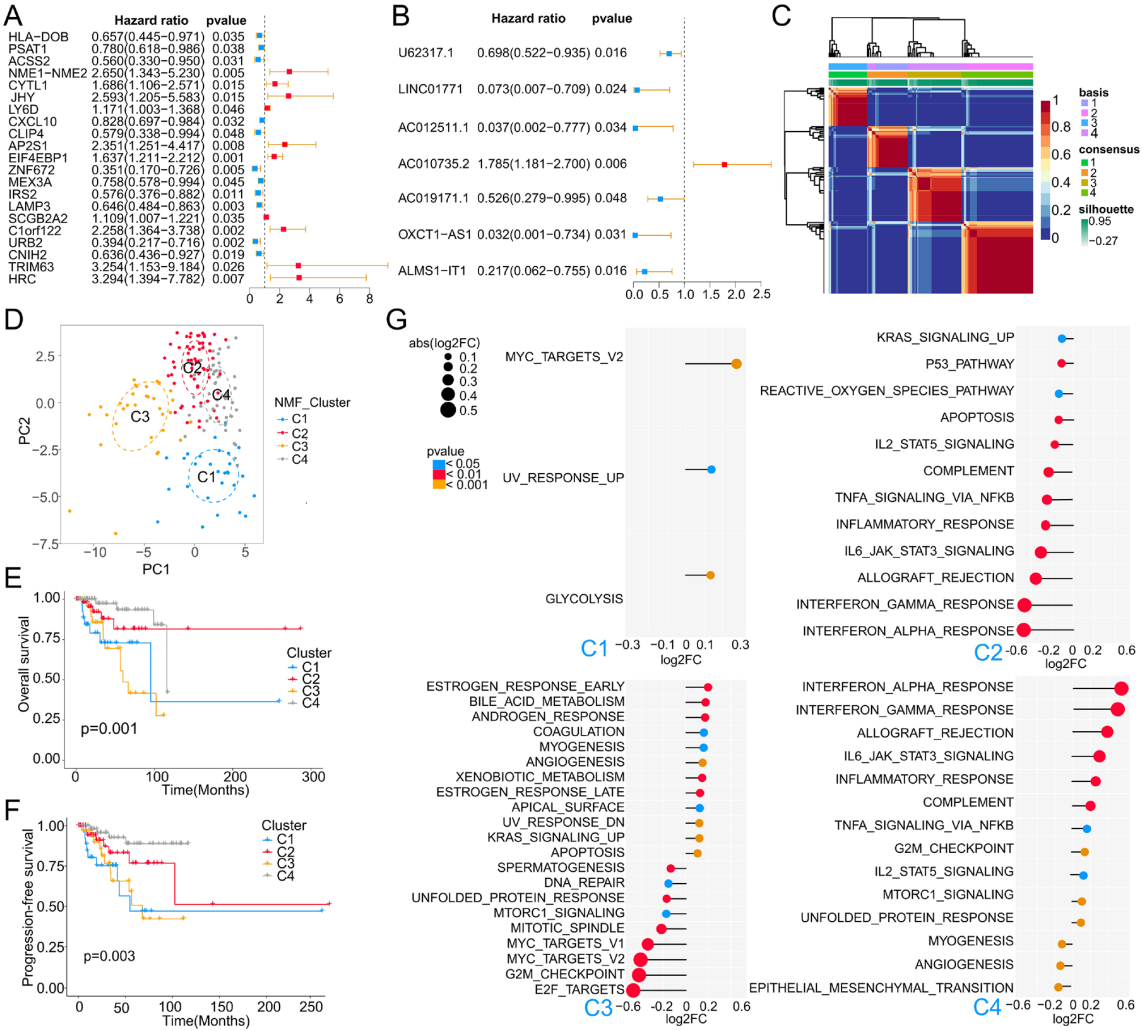
Identification of TNBC molecular subtype. (**A**,**B**) Univariate Cox analysis of mRNAs and lncRNAs, respectively. (**C**) Unsupervised clustering analysis of the TCGA-TNBC specimens using non-negative matrix factorization (NMF) and 23 BEZ235 response-related genes. An optimal rank of 4 was selected based on high cophenetic and silhouette coefficients (see Fig. S6). shown is the NMF matrix at rank of 4, and the subgroup assignments derived from this cluster solution are color-coded at top. (**D**) Principal component analysis (PCA) of TCGA-TNBC specimens using 23 BEZ235 response-related genes. (**E**,**F**) Kaplan–Meier overall survival (OS) curve and progression-free survival (PFS) curve for TCGA-TNBC patients of different clusters. (**G**) Based on the Hallmark gene set, GSVA analysis was utilized to mine the functional characteristics of each cluster relative to the other 3 clusters.

Based on TCGA-TNBC cohort, we performed a univariate Cox analysis of 451 DRRGs. As shown in Fig. 4A,B 21 DEmRNAs and 7 DElncRNAs had prognostic value (*P* < 0.05), while no DEmiRNAs were found to be significantly associated with patients' overall survival (OS). Then, the log-rank analysis was performed on the 28 DERNAs and Kaplan–Meier (K-M) survival curves are drawn for the RNAs with OS differences (*P* < 0.05), except for *TRIM63* (Figs. S4 and S5). RNAs undetected in GSE58812 and GSE135565 datasets were excluded. Eventually, 23 RNAs entered the next analysis, including *CXCL10*, *HRC*, *HLA-DOB*, *PSAT1*, *ACSS2*, *CYTL1*, *JHY*, *LY6D*, *CLIP4*, *AP2S1*, *EIF4EBP1*, *ZNF672*, *MEX3A*, *IRS2*, *LAMP3*, *SCGB2A2*, *C1orf122*, *URB2*, *CNIH2*, *LINC01771*, *ALMS1-IT1*, *AC010735.2*, and *U62317.1*.

To identify differences in the DRM of TNBC patients, we utilized 23 BEZ235 response-related genes associated with OS as typing markers. Interestingly, the TNBC samples were classified into four heterogeneous clusters (Fig. 4C). Our analysis revealed that four was the optimal and robust clustering number (Fig. S6), suggesting the existence of four drug response patterns. A PCA analysis further supported the heterogeneity of substantial intertumoral DRM (Fig. 4D), and classification into four clusters was found to be the most robust classification. K-M analysis confirmed significant differences in the OS and progression-free survival (PFS) among the four clusters (Fig. 4E,F). The cases in C4 and C2 clusters were associated with better OS and PFS. Importantly, Consensus clustering analysis was adopted and TNBC was also clustered into 4 clusters (Fig. S7A), which were almost identical to NMF clustering (Fig. S7B). Similarly, there were significant differences in OS and PFS among the four clusters (Fig. S7C,D). These results confirmed the DRM heterogeneity of TNBC and its prognostic significance.

The expression levels of 23 BEZ235 response-related genes in the four clusters are shown in Fig. S8A. We found that different clusters have their representative genes, which may be potential diagnostic indicators for typing. For example, the expression levels of *LY6D*, *EIF4EBP1*, *AP2S1* and *C1orf122* were higher in C1 than in other clusters. Then, GSVA analysis revealed distinct Hallmark pathways for each cluster relative to the others (Figs. 4G, S8B), and we defined four new molecular subtypes based on the characteristic pathways of each cluster. These four subtypes consisted of the following: C1 (glycolysis) subtype characterized by glycolysis pathway; C2 (proliferation depression) subtype characterized by downregulation of cell proliferation-related pathways and enriched in estrogen and androgen response, stroma formation (including angiogenesis and myogenesis), xenobiotic metabolism pathways; C3 (immune-suppressed) subtype characterized by downregulation of immune response-related signals; C4 (immune-activated) subtype with high immune response-related signals, while downregulation of angiogenesis, myogenesis and epithelial-mesenchymal transition signal. The results were correlated with the TNBC subtypes defined by Jiang^27^. In short, our results not only identified the existence of 4 molecular subtypes associated with DRM of TNBC, but also confirmed that BEZ235 response-related genes are potential novel biomarkers for molecular typing.

### Construction of drug response-related prognostic risk prediction model

**Figure 5:**
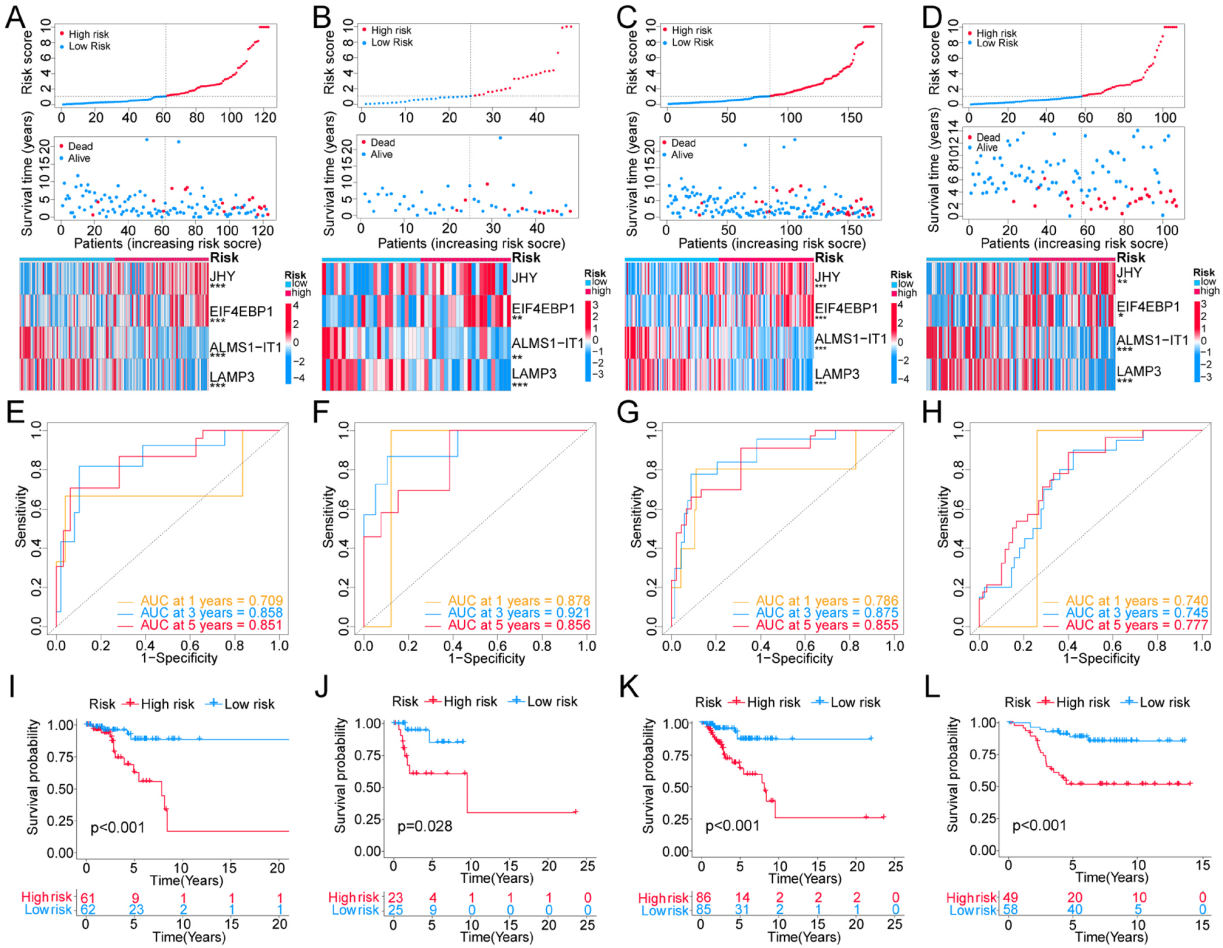
Evaluation of the Lasso–Cox model in four data sets. (**A**–**D**) The risk stratification diagram (including risk status map, risk point map and risk heat map) showed that patients in the high-risk group had worse overall survival (OS) in internal training set (**A**), internal validation set (**B**), entire TCGA-TNBC cohort (**C**) and GSE58812 cohort (**D**). (**E**–**H**) Time dependent ROC analysis of Lasso–Cox model in internal training set (**E**), internal validation set (**F**), entire TCGA-TNBC cohort (**G**) and GSE58812 cohort (**H**). (**I**–**L**) Kaplan–Meier survival analysis of Lasso–Cox model in internal training set (**I**), internal validation set (**J**), entire TCGA-TNBC cohort (**K**) and GSE58812 cohort (**L**). *P < 0.05; **P < 0.01; ***P < 0.001.

**Figure 6:**
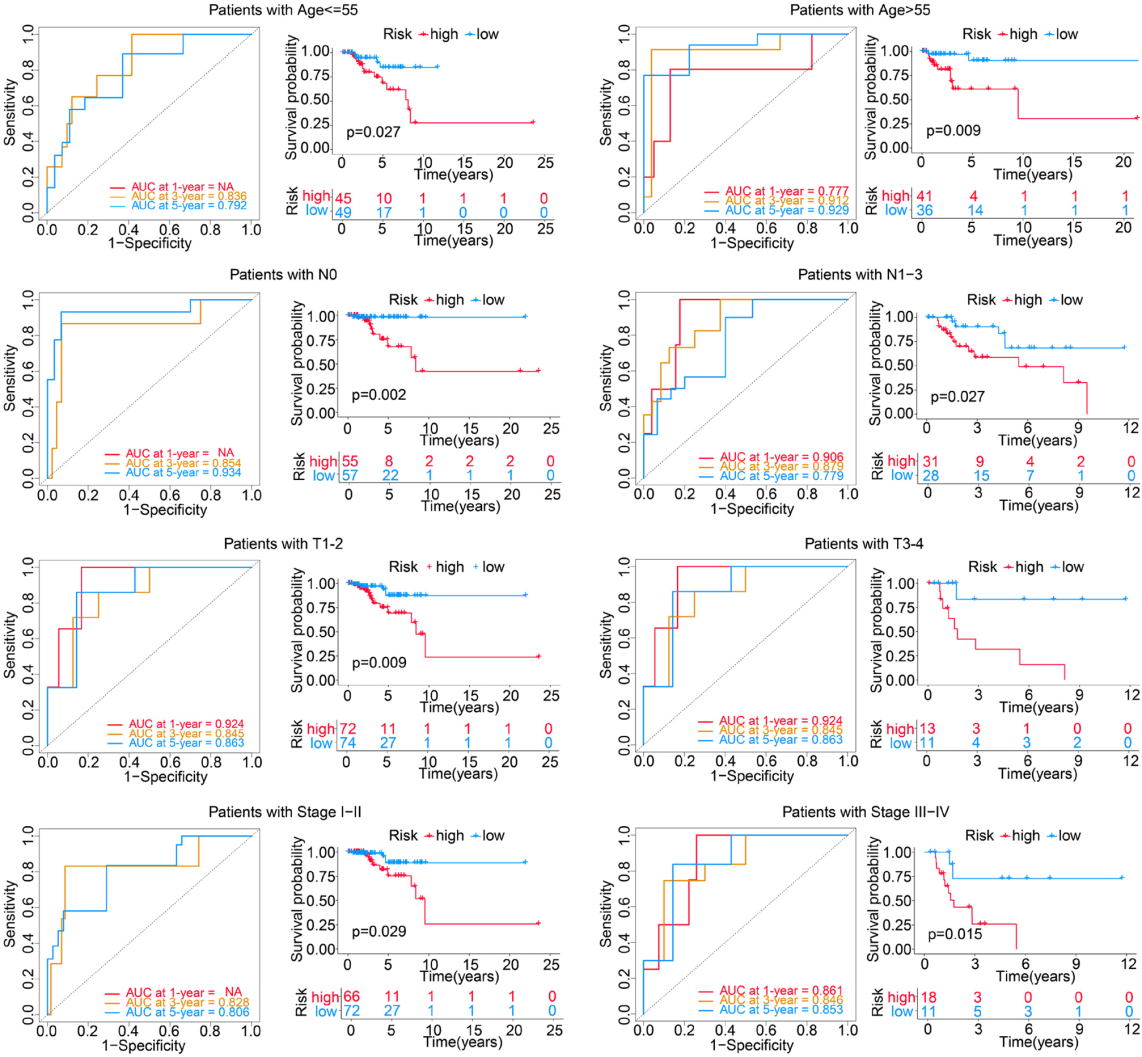
Time dependent ROC analysis and Kaplan–Meier survival analysis of the four-gene risk model in different clinical subgroups. NA: For less than two outcome events, no ROC curve was drawn.

Next, the 23 typed genes were subjected to Lasso–Cox regression analysis, and the model achieved the best performance with 4 genes in training set (Fig. S9). The Risk score (RS) of Lasso–Cox signature for OS was identified: RS = 2.122 * Exp *JHY* + 1.980 * Exp *EIF4EBP1* + (− 0.445) * Exp *ALMS1-IT1* + (− 0.473) * Exp *LAMP3*. Then, training set, validation set, entire TCGA cohort, and GSE58812 cohort, were separated to HRG and LRG based on the median of RS for model evaluation. Through risk stratification map (including risk state map, risk point map and risk heat map), it was found that the OS of TNBC patients in the death group was significantly shortened with the increase of the patient RS, and the HRG was associated with an increase in the number of death samples. The risk heat map showed the same expression trend of 4 genes among the four data sets, suggesting that the expression levels of these 4 genes were correlated with RS. Because we found that when the expression level of risk factors (*JHY* and *EIF4EBP1*) increased and the expression level of protective factors (*ALMS1-IT1* and *LAMP3*) decreased, the RS also increased, suggesting that patients with higher RS had a worse prognosis (Fig. 5A–D). As shown in Fig. 5E–H, the tdROC curve was analyzed and AUC values of 1-, 3- and 5-year were all greater than 0.70 in the 4 data sets, confirming the good diagnostic performance of the RS. The survival analysis demonstrated that the OS in low-risk group (LRG) was higher compared with the high-risk group (HRG) (Fig. 5I–L). In addition, it was found that AUC values of 1-, 3- and 5-year were all greater than 0.70 and the TNBC patients’ OS was still significantly different between the HRG and LRG in various clinical subgroups, including Age ≤ 55, Age > 55, T1-2, T3-4, N0, N1-3, Stage I-II and Stage III-IV (Fig. 6). Together these data indicated that our model performed robustly in different cohorts.

### The Lasso–Cox signature performed better than other reported signatures in prognostic prediction

**Figure 7:**
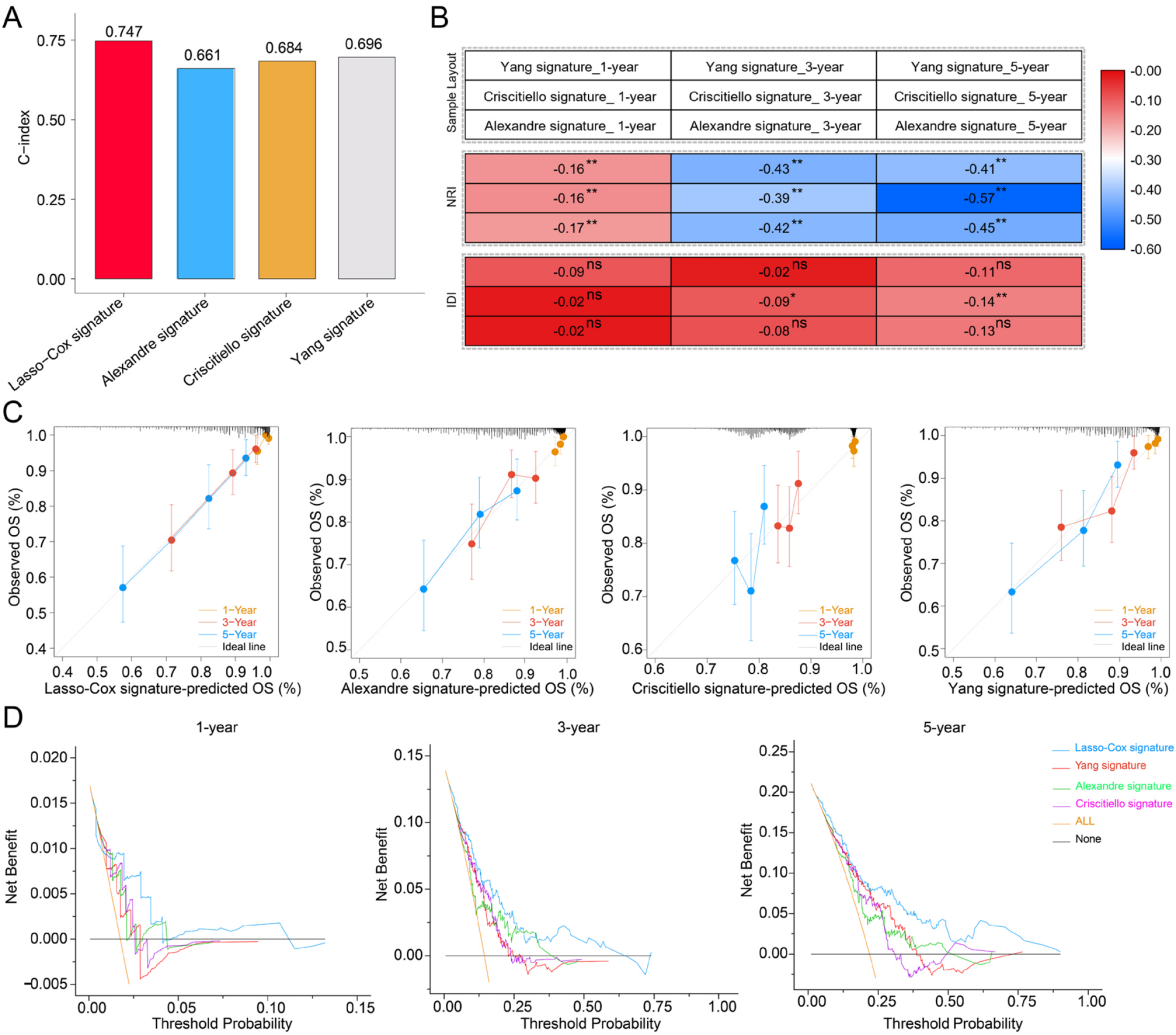
Evaluation of Lasso–Cox versus reported models in the integrated dataset (includig TCGA-TNBC, GSE58812 and GSE135561). (**A**) C-index analysis of the Lasso–Cox Signature, Criscitiello Signature, Alexandre Signature and Yang Signature. (**B**) INR and IDI analysis between reported models and Lasso–Cox model. (**C**,**D**) 1-year, 3-year and 5-year of calibration curve analysis and decision curve analysis (DCA) among Lasso–Cox Signature, Criscitiello Signature, Alexandre Signature and Yang Signature. *: *P* < 0.05; **: *P* < 0.01; ns: no significance.

Combined with literature mining, we selected 8 excellent reported models for comprehensive comparison with our model, including Li signature^28^, Peng signature^29^, Yang signature^30^, Yu signature^31^, Criscitiello signature^32^, Park signature^33^, Qin signature^34^ and Alexandre signature^35^. Three cohorts (TCGA-TNBC, GSE58812 and GSE135561) were merged as an integrated cohort for inter-model evaluation. Except for the Peng signature and Li signature, all other signatures could effectively divide patients into two subgroups with significantly different prognoses (*P* < 0.05) (Fig. S10). However, tdROC analysis revealed that the AUC values of the 8 external signatures for 1-, 3-, and 5-year survival were lower than those of our model (Fig. S11). Then, the signatures with AUC values greater than 0.650 at 1-, 3- and 5-year were selected for further analysis, including Lasso–Cox Signature, Criscitiello Signature, Alexandre Signature and Yang Signature. Immediately afterwards, the “restricted mean survival (RMS)” R package was used to calculate the C-index of the four prognostic signatures. Our model had the highest concordance index (C-index) at 0.747 (Fig. 7A). As shown in Fig. 7B, compared with our model, the reported three models decreased the relative , Net reclassification index (NRI), and our model significantly outperformed the Criscitiello signature model in the 3- and 5-year integrated discrimination improvement (IDI) evaluations. By Calibration curves, our model performed the best consistency between the actual survival probability and the predicted survival probability at 1-, 3- and 5-year (Fig. 7C). Decision curve analysis (DCA) was used to assess the validity of the signatures, our model had a higher overall net benefit compared to the reported models (Fig. 7D). To sum up, our model performed better than others in prognostic prediction.

### Performance of Lasso–Cox signature with regard to clinical features

To identify whether the RS could serve as an independent biomarker for prognosis, we performed univariate and multivariate Cox regression analyses of clinical data to assess the relevant hazard ratio (HR) and 95% confidence interval (CI) in the entire TCGA-TNBC dataset. The results revealed that the RS was an independent risk factor for prognosis (Table 1). Using the Sankey diagram, we showed the sample distribution and discovered that most of the C4 samples were from the LRG and showed alive status, while C1 and C3 were the opposite (Fig. S12A). Meanwhile, chi-square test also confirmed that there were significant differences in the risk sample distribution of the four subtypes (Fig. S12B). All these results again proved that C4 had the best prognosis, followed by C2, C3 and C1 had the worst prognosis, which was consistent with the previous survival analysis results. We evaluated the correlation between RS and clinicopathological parameters, and found that Stage III-IIV had a higher RS than Stage I-II (Fig. S12C–F). The above findings showed that the Lasso–Cox Signature has good predictive performance for the prognosis of patients in clinical application.

**Table 1:** The Cox regression analysis was performed to identify independent predictor of prognosis in TCGA-TNBC patients.

Variables	Univariate Cox analysis		Multivariate Cox analysis	
	HR (95%CI)	*P* value	HR (95%CI)	*P* value
Age	1.00 (0.97–1.03)	0.80	0.99 (0.96–1.02)	0.53
T stage	2.83 (1.73–4.63)	< 0.001	1.16 (0.59–2.29)	0.67
N stage	2.80 (1.89–4.14)	< 0.001	1.60 (0.83–3.06)	0.16
Stage	5.75 (2.96–11.16)	< 0.001	2.99 (0.90–9.88)	0.07
Risk score	1.12 (1.08–1.17)	< 0.001	1.11 (1.06–1.17)	< 0.001

### Function analysis in HRG and LRG

**Figure 8:**
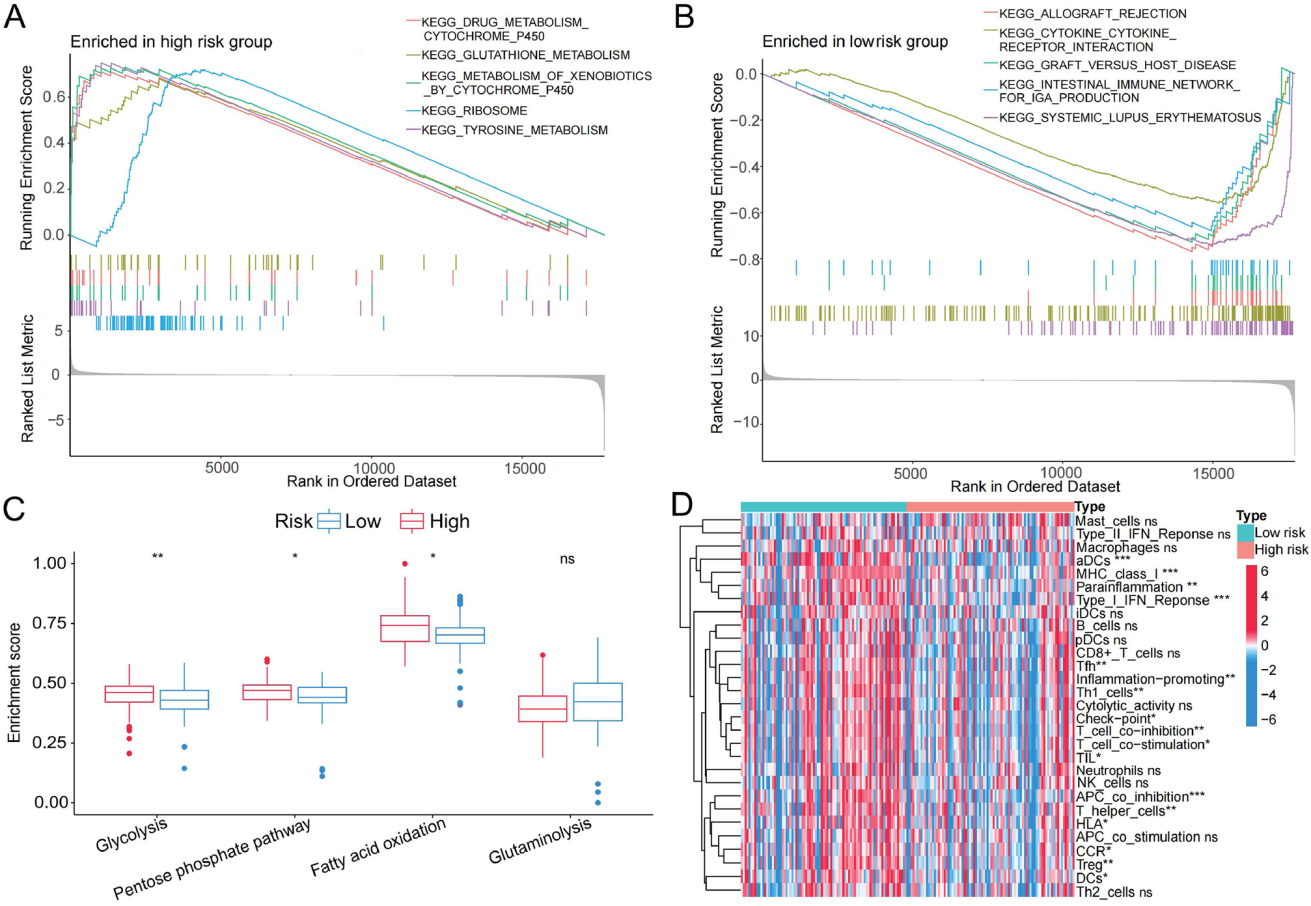
Functional enrichment analysis between high-risk group and low-risk group. (**A**) Top 10 of KEGG enrichment analysis showed that the low-risk group was enriched in pathways associated with immune full activation; (**B**) The high-risk group was prominently enriched in metabolism pathways. (**C**) Differential analysis of ssGSEA enrichment scores of four energy metabolism pathways between high- and low- risk group. (**D**) Differences in the ssGSEA enrichment scores of immune-related cells/pathways between high- and low-risk group in TCGA-TNBC cohort. *P < 0.05; **P < 0.01; ***P < 0.001; ns: no significance.

Based on TCGA-TNBC cohort, GSEA analysis was performed to understand pathways that were significantly enriched in the HRG and LRG. The top 5 enrichment results are shown in Fig. 8A,B. The KEGG enrichment analysis revealed that the LRG was enriched in pathways associated with inflammatory/immune activation, such as allograft rejection and inflammatory response. In comparison, the HRG was prominently related to multiple metabolism-related pathways, for instance drugs metabolism cytochrome P450. To confirm differences in the energy metabolism and immune activity between LRG and HRG, we calculated the ssGSEA scores to estimate the abundance of four metabolic pathway activities and the abundance of diverse immune signatures in each sample, respectively. We verified that the enrichment scores of glycolysis, pentose phosphate pathway (PPP) and fatty acid oxidation (FAO) pathways were significantly higher in the HRG (Fig. 8C). Moreover, diverse immune signatures, including tumor-infiltrating lymphocytes (TILs), check point, and the type I interferon (IFN) response, were increased in the LRG (Fig. 8D). These data highlight the RS as a potential indicator for identifying metabolic and immune infiltration characteristics in TNBC patients.

### Response of high- and low-risk patients to immunotherapy and chemotherapy

**Figure 9:**
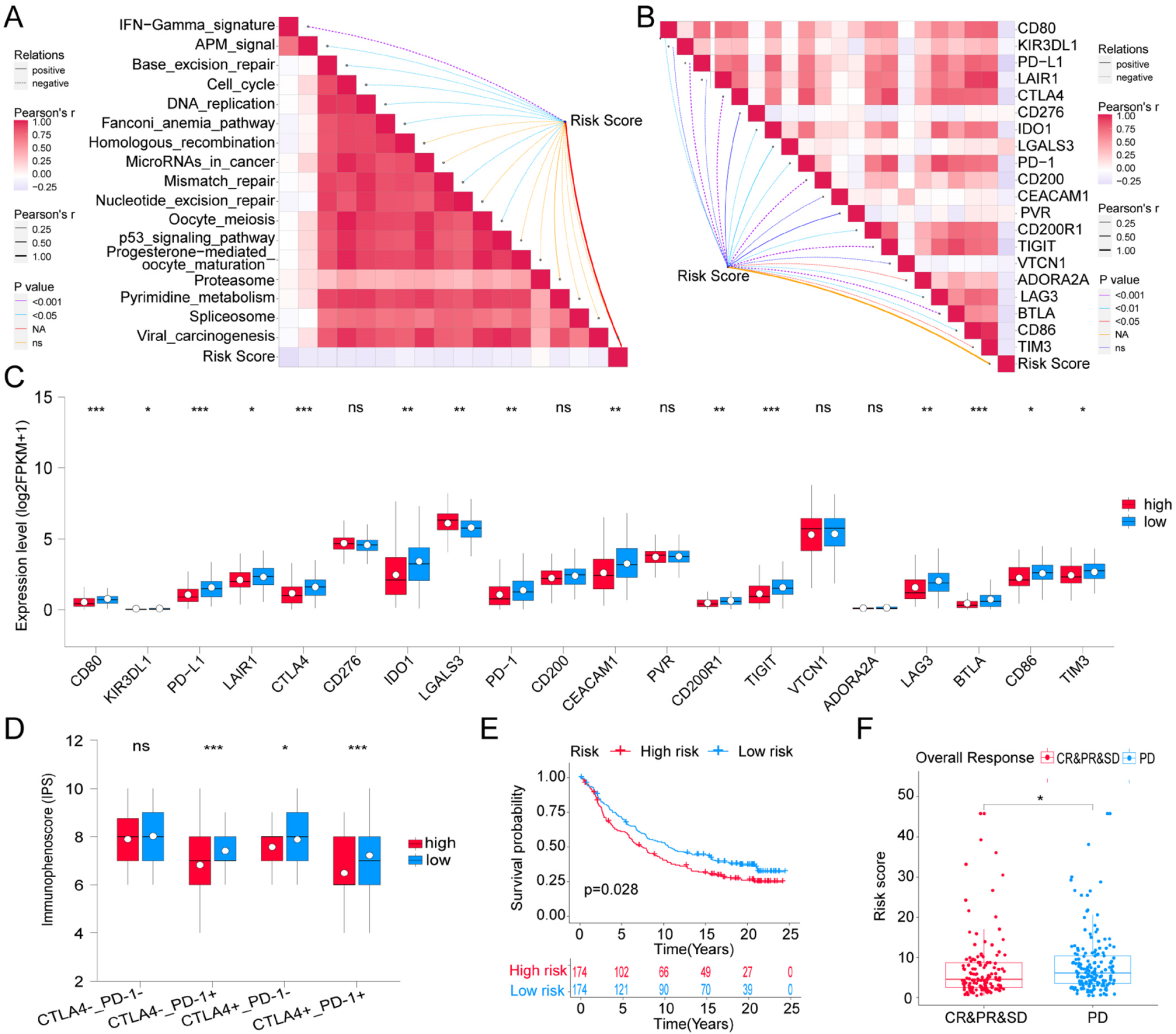
Roles of Risk score in predicting immune phenotypes in TCGA-TNBC cohort. (**A**) Correlations between Risk score and the enrichment scores of immunotherapy-predicted pathways. (**B**) Correlations between Risk score and immune checkpoints. (**C**) Analysis of differential expression level of immune checkpoints between high- and low-risk groups in TCGA-TNBC cohort. (**D**) The association between immunophenoscore (IPS) and the Risk score of TNBC patients in TCGA. (**E**) Kaplan–Meier survival analysis of Lasso–Cox Signature in IMvigor210 cohort for overall survival. (**F**) The difference of Risk score in the subgroup of PD-1 treatment response in IMvigor210 cohort. *P < 0.05; **P < 0.01; ***P < 0.001; ns: no significance; NA: not applicable.

In theory, patients with lower RS should have a higher response to Immune Checkpoint Blocker (ICB) because low-RS defines an immune response-enriched tumor microenvironment (TME). A series of potentially targetable immune checkpoint genes that were designed for inhibitors, and many immunotherapy-related signatures can guide the immunotherapy choice for TNBC patients. Here, we first analyzed the correlations between RS and various immune signatures. As expected, the RS negatively correlated with the enrichment scores of several immunotherapy-positive gene signatures (Fig. 9A) and the expression levels of most inhibitory immune checkpoints (Fig. 9B). Similarly, difference analysis further showed that most of these inhibitory immune checkpoints were up-regulated in LRG inclined to immune activation phenotype (*P* < 0.05), including PD-1, PD-L1 and CTLA4 (Fig. 9C). Furthermore, we used the immunophenoscore (IPS) to assess the potential of ICB application for TNBC patients. Unsurprisingly, all other IPSs were significantly higher in LRG compared to HRG except IPS_CTLA4-_PD1- (*P* < 0.05) (Fig. 9D). The results suggested that the TNBC patients in the LRG had a better opportunity for ICB application. To further confirm that the RS might serve as a predictor for immunotherapy response, the IMvigor210 cohort including 348 urothelial carcinoma patients who received immunotherapy were enrolled for analysis^36^. Consistent with the previous results, the LRG presented with better survival (*P* = 0.028) (Fig. 9E). At the same time, the higher RS was observed in the progression response group (Fig. 9F). The results validate the conclusion that RS might serve as an indicator for immunotherapy.

**Figure 10:**
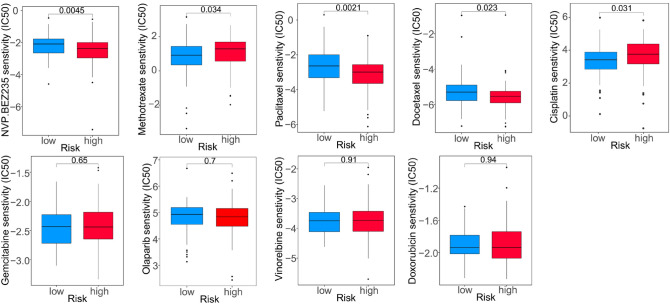
Evaluation of chemosensitivity by the risk model.

Finally, the pRRophetic algorithm was used to predict the IC50 of one targeted therapeutic agent (BEZ235) and 8 common chemotherapeutic agents (cisplation, methotrexate, docetaxel, paclitaxel, olaparib, gemcitabine, vinorelbine and doxorubicin) in high- and low-risk patients in TCGA-TNBC cohort. We observed that patients in the HRG were more sensitive to BEZ235, docetaxel and paclitaxel, and in the LRG were more sensitive to methotrexate and cisplation (Fig. 10). In conclusion, our results indicated that RS also might serve as an indicator for chemotherapy.

## Discussion

Because of the heterogeneity and the absence of well-defined molecular targets, the treatment options and prognosis management of TNBC remain a clinical challenge. Studies on the role of BEZ235 in TNBC have gradually proved that it is a potential drug for TNBC patients. Abnormal gene expression is closely associated with the response to therapy of TNBC patients. However, the role of BEZ235 response-associated genes in TNBC has not been explored. Here, by simulating the DRM of TNBC patients treated with BEZ235, multidimensional transcriptomic data of cell lines and primary tumor tissues were combined to obtain BEZ235 response-related genes. Then, we performed a panoramic analysis of BEZ235-responsive genes. First, by constructing a BEZ2335 response-related ceRNA network, the anti-tumor mechanism of BEZ235 was confirmed and the adaptive changes of tumor cells under conditions detrimental to survival were investigated. Second, we identified four molecular subtypes associated with the drug-responsive microenvironment in TNBC. Finally, an excellent prognostic risk prediction model was constructed in TNBC. To our best knowledge, this is the first report to systematically and comprehensively confirm the role of BEZ235 response-related genes in TNBC, which not only deepens the understanding of the BEZ235 pharmacological mechanism, but also provides a reliable experimental basis for in-depth research on TNBC.

We presented and discussed the following new findings:

First, we constructed the drug response-related ceRNA networks in MDA-MB-468 cells including a subnetwork closely related to BEZ235 antitumor mechanisms and a subnetwork related to BEZ235-induced cellular resistance programs. The regulatory directions of all lncRNA-miRNA-mRNA axes in the network were opposite in the TCGA-TNBC tissues, indicating that these regulatory axes play an extremely important role in the anti-tumor mechanism of BEZ235. Furthermore, we found that the down-regulated DEmRNAs except SRM all came from the mTORC1 signaling pathway and targeted by *has-miR-143-3p*, including *SLC7A5*, *SLC7A11*, *TUBG1*, *GPI*, *HMBS*, *PNP*, *PDAP1*, *CDC25A* and *PSMC4*. Notably, Yothaisong et al. had verified that BEZ235 caused a reduction in *SLC7A5* expression in parallel with a reduction of activated AKT and mTOR in cholangiocarcinoma cell^37^. Numerous studies have confirmed that the miR-143-3p/SLC7A11 axis is involved in various tumor progression^38,39^. Baumann et al. had confirmed that BEZ235 acted on myeloma cells and induced downregulation of *CDC25A*, which might be involved in cell cycle G1 phase arrest, thereby inhibiting cell proliferation^40^. Our data for the first time proved that BEZ235 mainly acted on mTORC1 signaling pathway to exert an anti-tumor effect from the perspective of ceRNA regulation, which was consistent with the previous study^22^. Li et al. had proved that *hsa-miR-143-3p* targeting LIM domain kinase 1 suppresses the progression of TNBC cells^23^. Therefore, the ceRNA network with *hsa-miR-143-3p* as the core may be the potential therapeutic target for TNBC.

It was worth noting that in the ceRNA network with miRNAs down-regulated as the core, a large number of genes had been confirmed to be involved in mechanisms of drug sensitivity or resistance in BC^41–46^. For example, Minemura et al. demonstrated that upregulation of *CITED2* expression caused chemoresistance to epirubicin and 5-fluorouracil in MCF7 and SK-BR-3 cell^47^. Liu et al. found that ATF3 regulated BC cell resistance to tamoxifen through an N6-adenosine-based epigenetic transcriptomic mechanism and suggested that *ATF3* may be a candidate therapeutic target to overcome drug resistance in cancer cell^48^. Importantly, Simpson et al. found that the upregulation of IRS2 mRNA and protein in MDA-MB-468 cells was dependent on PI3K inhibition^24^. They proposed that IRS2 may be oncogenic and that PI3K inhibitors lead to the activation of a feedback loop involving IRS2, in which cells attempt to bypass apoptosis and growth inhibition through PI3K up-regulation signaling. In addition, Guan et al. verified that *hsa-miR-33a-5p* overexpression made TNBC sensitive to doxorubicin by inhibiting eIF5A2 expression and epithelial-mesenchymal transformation^49^. Kim et al. found that the overexpression of miR-500a-3p sensitized ERα-negative cells to tamoxifen by increasing apoptosis^50^. Tan et al. confirmed that the dynamically decreased miR-671-5p expression was associated with oncogenic transformation and radiochemoresistance in BC^51^. In conclusion, the multiple resistance genes upregulated or sensitivity genes downregulated in this subnetwork may be one of the potential factors for the poor efficacy of BEZ235 monotherapy. In the future, the development of therapeutic drugs targeting these targets is expected to improve the poor efficacy of BEZ235 monotherapy.

Second, molecular typing based on transcriptome data has proven to be an effective way to organize tumor heterogeneity. This study identified four molecular subtypes closely related to the with significant differences in OS and PFS, including glycolysis subtype, proliferation inhibition subtype, immunosuppression subtype and immunoactivation subtype, which were significantly similar to the molecular subtype proposed by Jiang et al.^27^. Thereinto, the immune-activated subtype had the best prognosis compared to the others. We first proposed two new subtypes in TNBC, including the glycolysis subtype characterized by glycolysis pathway, and proliferative depression subtype characterized by downregulation of cell proliferation-related pathways. Glycolysis subtype may represent the cluster of tumor cells that undergo metabolic reprogramming after drug treatment. It is worth mentioning that Yu et al. found that basal-like BC was dominated by glycolysis and PPP metabolism, which implied the existence of this subtype^52^. In addition, cytotoxic and targeted therapies have been shown to drive cells into drug tolerant persister (DTP) cell states that can survive drug pressure in a low-proliferative state, leading to incomplete response and/or recurrence^53,54^. Thus, the proliferative depression type identified in this study is a new potential subtype of TNBC. In a word, molecular typing based on DRRGs proposed in this study was expected to guide drug therapy for TNBC patients. Soon, more drugs with different mechanisms of action are needed to be incorporated into the molecular typing of tumor patients, to establish a unified clinical typing index and participate in the management of drug therapy for clinical patients.

Third, we constructed a Lasso–Cox model, which was composed of 3 mRNAs (*JHY*, *EIF4EBP1* and *LAMP3*) and 1 lncRNA (*ALMS1-IT1*). We found that these four genes have been reported in multiple prognostic models, for example, *EIF4EBP1* was involved in the construction of autophagy-related prognostic model to predict the prognosis of multiple myeloma^55^, breast cancer^56,57^ and endometrial cancer^58^. *EIF4EBP1* also acts as a redox-related gene involved in constituting a prognostic marker for clear cell renal cell carcinoma^59^. *ALMS1-IT1* was involved in constructing a ferroptosis-related prognostic model for predicting the prognosis of colorectal cancer^60^, and a risk model of 4 lncRNAs for predicting the survival time of patients with head and neck squamous cell carcinoma^61^. In addition, multiple studies had shown that *LAMP3*, as an immune/inflammation-related gene, was involved in the construction of prognostic models and could well predict the prognosis of lung adenocarcinoma^62,63^. Therewith, a single model assessment was performed using risk stratification graph, tdROC and K-M analysis, it is confirmed that our model has high diagnostic accuracy and discrimination ability in four data sets. In order to fully evaluate the performance of the prediction model, we mined 8 previous reported models for comparative analysis. tdROC curve and C-index also showed our model was a good performance in prognosis prediction than the others in an integrated dataset. Compared with our model, the reported models decreased the relative NRI and IDI. The calibration curve and DCA confirmed that our model had the best predictive consistency and the greatest clinical benefit. These findings highlighted the superior predictive performance of our model. Thus, it may be useful during the clinical decision-making process and for choosing individualized treatments.

Fourth, the function analysis revealed that the LRG was enriched in pathways associated with immune activation. The HRG was prominently related to multiple metabolism-related pathways, including drug metabolism and glycolysis. Furthermore, the RS negatively correlated with the enrichment scores of many immunotherapy-positive gene signatures and the expression levels of most immune checkpoint inhibitors. These results indicated that the lower RS predicts a better immunotherapy response. Moreover, the IPS and IMvigor210 cohort analysis suggested that the TNBC patients in the LRG may benefit more from immunotherapy than those in the HRG. Meanwhile, sensitivity analysis revealed that the patients with high RS was associated with high sensitivity of cisplatin and methotrexate, and the patients with low RS was significantly more sensitive to BEZ235, docetaxel and paclitaxel. In conclusion, these considerations can guide the clinical selection of immunotherapy and chemotherapy drugs.

## Conclusion

Our study not only provides a research basis for typing, prognosis and treatment of TNBC, but also opens up a new direction for the value of DRRGs in tumor research. However, this study is mainly based on bioinformatics speculation, which needs to be confirmed by further basic experiments.

## Materials and methods

### Cell culture

Human TNBC cell lines MDA-MB-231 (p53R280K) and MDA-MB-468 (p53R273H) were purchased from Cell Bank in Chinese Academy of Sciences in Shanghai. MDA-MB-231 was maintained in DMEM, whereas MDA-MB-468 Cells were cultured in Leibovitz’s L-15 supplemented with 10% FBS (HyClone; GE Healthcare Life Sciences, Logan, UT, USA), 100 U/mL penicillin and 100 μg/mL streptomycin at 37 °C with 5% CO_2_. When growth reached 80% confluence, MDA-MB-231 and MDA-MB-468 were treated in triplicate with 2 μM and 0.2 μM BEZ235, respectively, with 0.01% DMSO control^20^, which were incubated for 48 h and 72 h, respectively. The cells were removed as raw materials for subsequent RNA analysis.

### Transcriptome high-throughput analysis of MDA-MB-468

Total RNA from MDA-MB-468 cells was used for transcriptome analysis. The microarray hybridization and miRNA sequencing were performed by Kangchen Bio-tech (Shanghai P.R. China). Microarray analysis was performed using Arraystar Human LncRNA Microarray V4.0, which was designed for the global profiling of human lncRNA and mRNA. The miRNA was sequenced on Illumina NextSeq 500 sequencer and was quantified using miRDeep2.

### Differential RNA expression analysis

The Cancer Genome Atlas (TCGA, https://portal.gdc.cancer.gov/) data: Available mRNA and lncRNA sequencing data (including 173 TNBC samples and 113 breast normal samples) and miRNA isoform data (including 159 TNBC samples and 103 breast normal samples) were downloaded via the TCGA data portal. Gene Expression Omnibus (GEO, http://www.ncbi.nlm.nih.gov/geo/): Two TNBC GEO cohorts with detailed survival data were downloaded, namely GSE58812 (107 TNBC samples) and GSE135565 (84 TNBC samples). The expression value of these samples was detected using the Affymetrix Human Genome U133 Plus 2.0 Array platform. In addition, based on the Creative Commons 3.0 License, the complete expression data and detailed clinical information of the IMvigor210 cohort (a bladder cancer immunotherapy-related cohort) were obtained from http://research-pub.Gene.com/imvigor210corebiologies/.

DEmRNAs, DElncRNAs and DEmiRNAs were identified on TCGA-TNBC tissues vs. normal tissues and BEZ235-treated cells vs. controls. We use the “limma” package and “edgeR” package in software R to perform DEmRNAs/lncRNAs identification and DEmiRNAs analysis, respectively. |log_2_FC| ≥ 1 and *P*_*FDR*_ < 0.05 were used for selecting DEmRNAs and DElncRNAs. For DEmiRNAs, the select indicator was |log_2_FC| ≥ 0.5 and the *P*_*FDR*_ < 0.05.

### Weighted gene co-expression network analysis (WGCNA) and functional enrichment analysis

The WGCNA was conducted to identify the hub genes and modules associated with tumor trait in TCGA-TNBC dataset. The “WGCNA” package (http://www.r-project.org/) was used for WGCNA installation, data reading and import in R software^64^. The goodSamplesGenes function was used to determine whether the sample data were complete. The method was also used for sample clustering to identify and remove outliers. Pearson correlation coefficients between each group of genes were also calculated, and their absolute values were used to construct the gene expression similarity matrix according to the following formula: ${\text{a}}{}_{{{\text{ij}}}} = \left| {{\text{cor}}\;\left( {{\text{x}}_{{\text{i}}} ,{\text{x}}_{{\text{j}}} } \right)} \right|^{\upbeta }$, where x_i_ and x_j_ represent nodes i and j of the network, respectively. The adjacent matrix was constructed by choosing appropriate β values to make the gene distribution conform to the connection-based scale-free network. Then, the adjacency matrix and topological overlap matrix (TOM) were constructed. The TOM obtained was clustered by dissimilarity between genes, and the trees were cut into different modules by the dynamic shear method. The minimum number of genes was set as 50 for each module, and the module with closer distances was merged into a new module according to the threshold of 0.25. Gene significance (GS) was computed to evaluate the correlation between sample traits and genes. The correlations between modules and sample traits were defined as module significance (MS) via computing the average absolute GS of genes from the relevant modules. Ultimately, the MS ≥ 0.60, *P* < 0.05 and GS ≥ 0.3,* P* < 0.05 were set as the criteria to identify hub genes and modules.

### Construction of ceRNA network

The gene set “h.all.v7.4.symbols” was downloaded in gmt format from Molecular Signatures Database (MSigDB)^65^ (https://www.gsea-msigdb.org/gsea/msigdb/index.jsp) for gene set variation analysis (GSVA) (GSVA function in R). We adopted the lmFit analysis of the “limma” package to obtain the differential pathways between TCGA-TNBC tissues and normal tissues, and between BEZ235-treated cells and controls. Then, we extracted the BEZ235 response-related DEmRNAs in the differential pathways as candidates to participate in the construction of ceRNA network. Multiple databases or softwares were used to predict miRNA-mRNA and miRNA-lncRNA relationship pairs. The RNAhybrid (https://bibiserv.cebitec.uni-bielefeld.de/rnahybrid/), miRand (http://www.microrna.org/microrna/home.do), TargetScan (http://www.targetscan.org/), miRcode (http://www.mircode.org/index.php), miRDB (http://www.mirdb.org/custom.html), TarBase (v8, http://www.microrna.gr/tarbase), miRTarBase (http://miRTarBase.cuhk.edu.cn/) and starBase (http://starbase.sysu.edu.cn/) databases were performed to build the miRNA-mRNA relationship pairs. The miRanda, RNAhybrid, TargetScan, miRcode, lncBase (v3, www.microrna.gr/LncBase) and starBase were used to predict the lncRNA-miRNA relationship pairs. The generated networks were visualized by Cytoscape software (v3.8.2, https://www.cytoscape.org/).

Filtering criteria for the relationship pairs: ① Only mRNAs and lncRNAs in the opposite direction with miRNA regulation were selected to improve the accuracy of prediction results. ② The same relationship pair from at least three prediction type databases (miRanda, RNAhybrid, TargetScan, miRcode and miRDB) was selected. ③ When the same relationship pair came from two databases, at least one of them was a database of experimental verification type (Tarbase, miRTarBase, lncBase and starBase). ④ The miRNA-mRNA and miRNA-lncRNA pairs sharing common miRNA were included in the ceRNA network. The generated networks were visualized by Cytoscape software (v3.8.2, https://www.cytoscape.org/).

### Real-time quantitative PCR (RT-qPCR)

Total RNA was extracted from 15 pairs of 10 μm-thick formalin-fixed, paraffin-embedded TNBC and adjacent normal tissue scrolls using the paraffin-embedded tissue microRNA Rapid Extraction Kit (Bioteke Corporation, China) as per the manufacturer’s instructions. All patients were confirmed with primary TNBC by pathological examination of surgical specimens and were not subject to any preoperative radiotherapy or chemotherapy, other malignant diseases, or acute injury. Written informed consent was obtained from all patients prior to sample collection. The study was approved by the Ethical Review Committee of the Affiliated Hospital of Southwest Medical University (Luzhou, China). Total RNA of BEZ235-treated and MDSO-treated MDA-MB-468 cell lines was derived from previous MDA-MB-468 RNA sequencing materials.

The reverse transcriptional analysis of mRNA/lncRNA and miRNA was performed using PrimeScript™ RT reagent Kit with gDNA Eraser (TaKaRa, Japan) and Mir-X miRNA First-Strand Synthesis Kit (TaKaRa, Japan), respectively, according to the manufacturer's protocols. The expression level of the RNA was detected by RT-qPCR using TB Green™ Premix Ex Taq™ II (TaKaRa, Japan) in ABI Stepone plus (USA). GAPDH and U6 served as an control of mRNA/lncRNA and miRNA, respectively. The relative expression of RNAs were calculated using the 2^−ΔCq^ method. Primer sequences were designed by Primer Premier 6.0 and synthesized by Sangon Biotech (Shanghai,China) (Table S4).

### Cluster analysis

By performing non-negative matrix factorization (NMF) analysis with the “brunet” method for 50 iterations, we clustered the TCGA-TNBC cohort. The clustering number k was set as 2 to 10, and we further determined the average profile width of common member matrix by using “NMF” package in R with the minimum member numbers of each subclass set to 10. According to indexes including cophenetic and silhouette, the optimal number of clusters was finally determined. Concurrently, consensus clustering (ConsensusClusterPlus function in R) was performed to further verify the optimal number of clusters using 1000 iterations.

### Lasso–Cox regression analysis

TCGA-TNBC cohort was divided into training set and validation sets with a ratio of 7:3. GSE58812, as testing set, was used to test model stability. In the training set, the Lasso algorithm was applied to screen optimal candidate RNAs with the best discriminative capability. We then developed a RS based on the RNA-expression profiles using the “glmnet” R package, weighted using the multivariate Cox regression coefficient as follows:

$$RS={\beta }_{1}*Exp{X}_{1}+{\beta }_{2}*Exp{X}_{2}+\cdots \cdots +{\beta }_{N}*Exp{X}_{N}$$

N is the number of selected genes, X was the selected gene, Exp was the gene expression value, and β was the risk coefficient of each factor calculated by the Lasso–Cox model. This information was used to classify patients into two groups: LR and HRG. Next, we used a Kaplan–Meier analysis to determine the OS among the two risk groups.

### Calculation of the enrichment scores of various gene signatures

The “c2.cp.kegg.v7.4.symbols” from MSigDB was used for gene set enrichment analysis (GSEA) using the “clusterProfiler” R package between HRG and LRG. Four metabolic gene sets including glycolysis, PPP, FAO and glutaminolysis were collected from the previous study^52^, as well as the immune gene set involving many different types of human immune cell subtypes and immune-related activities^66,67^, for evaluation of metabolic- and immune-related characteristics in TME. Moreover, the targeted immunotherapy-associated gene signatures from the study of Hu et al.^68^ and 20 inhibitory immune checkpoints with therapeutic potential from Auslander’s study^69^, were collected for evaluation of immunotherapy-related characteristics.

### Drug sensitivity analysis

The IPS was calculated with a range of 0–10 based on the z-score for gene expression of representative cell types, and used to predict immune checkpoint response inhibitors of PD-1 and CTLA4 in the LRG and HRG. The IPS for TNBC patients was downloaded from the Cancer Immunome Atlas (TCIA, https://tcia.at/home). “pRRophetic” R package was used to predict IC50 of commonly used chemotherapy drugs in each sample^70^. IC50 indicates the effectiveness of a substance in inhibiting specific biological or biochemical functions.

### Statistical analysis

R software (v4.04, https://www.r-project.org) was used to analyze all data and generate the corresponding figures. tdROC curve, C-index, DCA, NRI and IDI were applied to model evaluation. The continuous variables fitting a normal distribution between binary groups were compared using a t-test. Otherwise, the Mann–Whitney U test was applied. Categorical variables were compared using the chi-squared test or Fisher’s exact test. Correlations between variables were explored using Pearson coefficients. The level of significance was set at *P* < 0.05, and all statistical tests were two-sided.

## Supplementary Information

Supplementary Figures.

Supplementary Table S1.

Supplementary Table S2.

Supplementary Table S3.

Supplementary Table S4.

## Data Availability

Publicly available datasets analyzed during the current study can be found here: TCGA, https://portal.gdc.cancer.gov/; GEO, http://www.ncbi.nlm.nih.gov/geo/; IMvigor210 cohort, http://research-pub.Gene.com/imvigor210corebiologies/. The multidimensional transcriptome dataset of MDA-MB-468 cell line during the current study are not publicly available due to the relevant article unpublished, but is available from the corresponding author on reasonable request. All methods were carried out in accordance with relevant guidelines and regulations in the manuscript.

## Abbreviations

BEZ235: NVP-BEZ235
DRRGs: Drug response-related genes
TNBC: Triple-negative breast cancer
DRM: Drug response microenvironment
DEmRNAs/lncRNAs/miRNAs: Differentially expressed mRNAs/lncRNAs/miRNAs
OS: Overall survival
PFS: Progression-free survival
K-M: Kaplan Meier
RS: Risk score
tdROC: Time dependent ROC
LRG/HRG: Low/high-risk group
RMS: Restricted mean survival
GS: Gene significance
MS: Module significance
GSEA: Gene set enrichment analysis
PPP: Pentose phosphate pathway
FAO: Fatty acid oxidation
TME: Tumor microenvironment
IPS: Immunophenoscore
C-index: Concordance index
DCA: Decision curve analysis
NRI: Net reclassification index
IDI: Integrated discrimination improvement
