# Supplementary material for: Identification and panoramic analysis of drug response-related genes in triple negative breast cancer using as an example NVP-BEZ235
Source: Sci Rep. 2023 Apr 12;13:5984. doi: 10.1038/s41598-023-32757-4 (PMC10097725; doi:10.1038/s41598-023-32757-4)
Supplement: Supplementary file 1 — Supplementary Figures. [file 41598_2023_32757_MOESM1_ESM.pdf]

- **Supplementary File**

Supplementary Figures

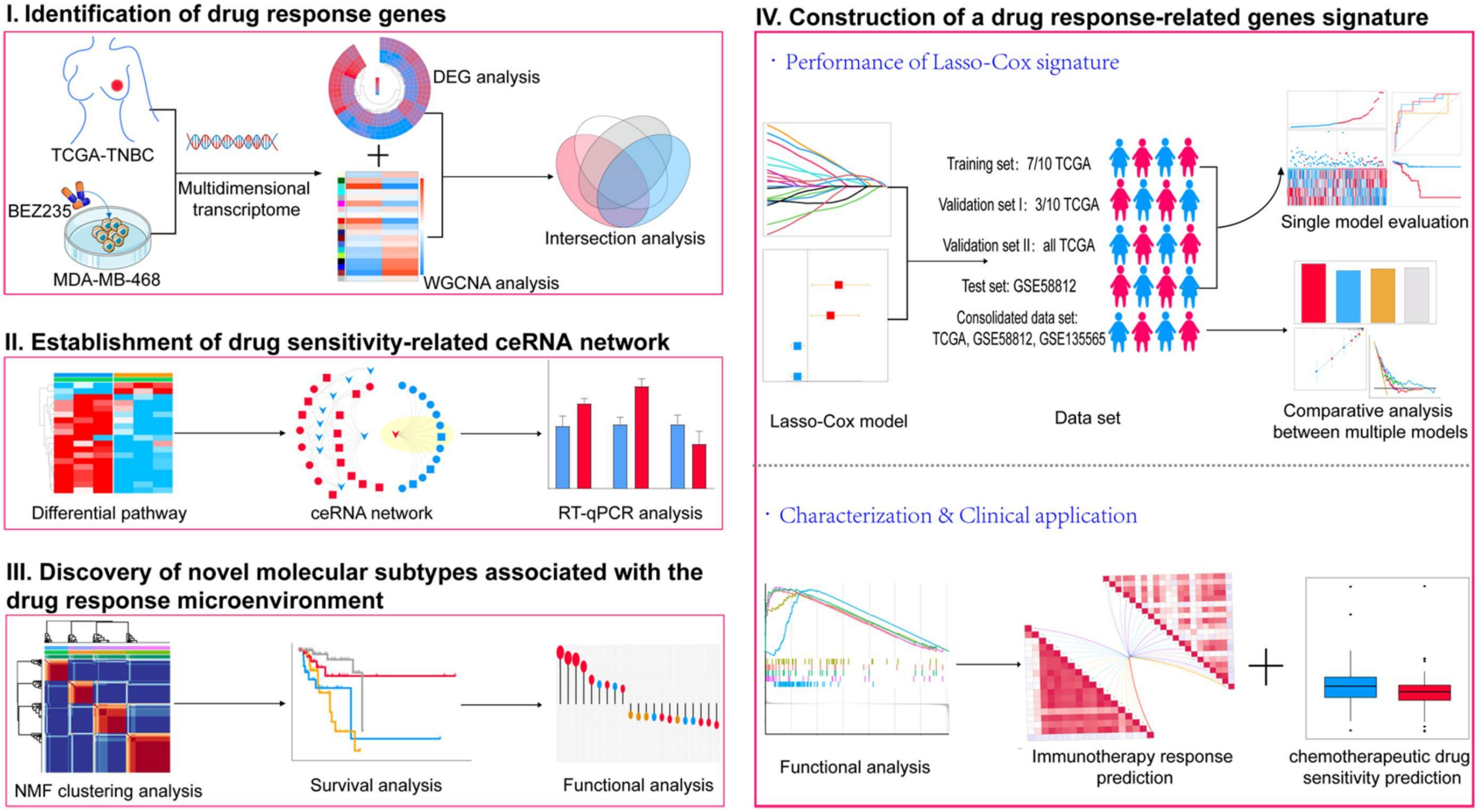

Figure S1 The flow chart of this study.

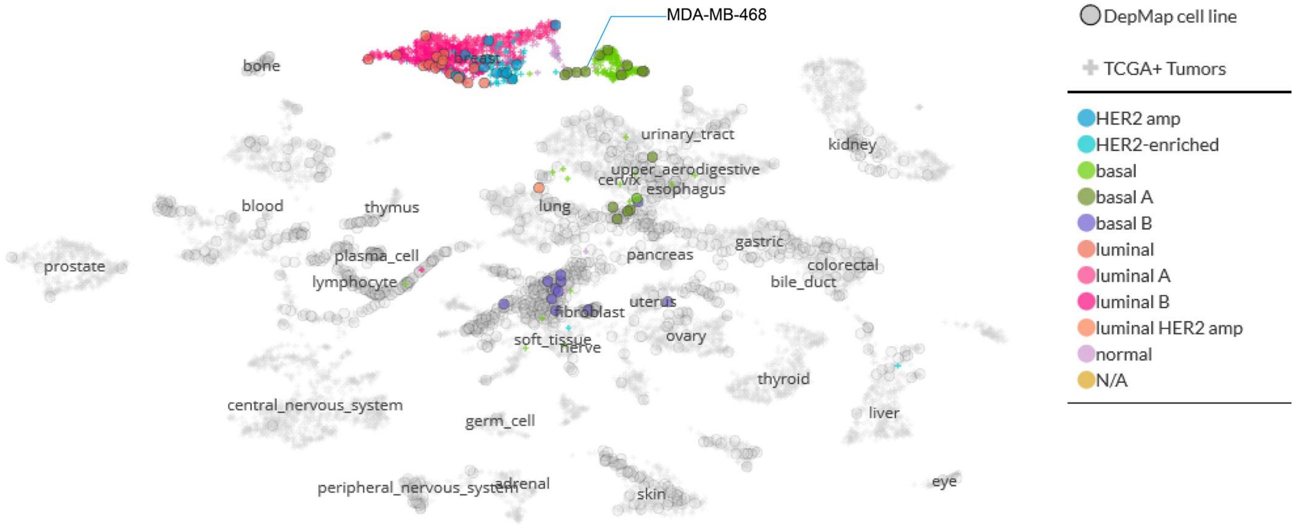

Figure S2 Transcriptome fidelity assessment of MDA-MB-468. (Downloaded from https://depmap.org/portal/celligner/)

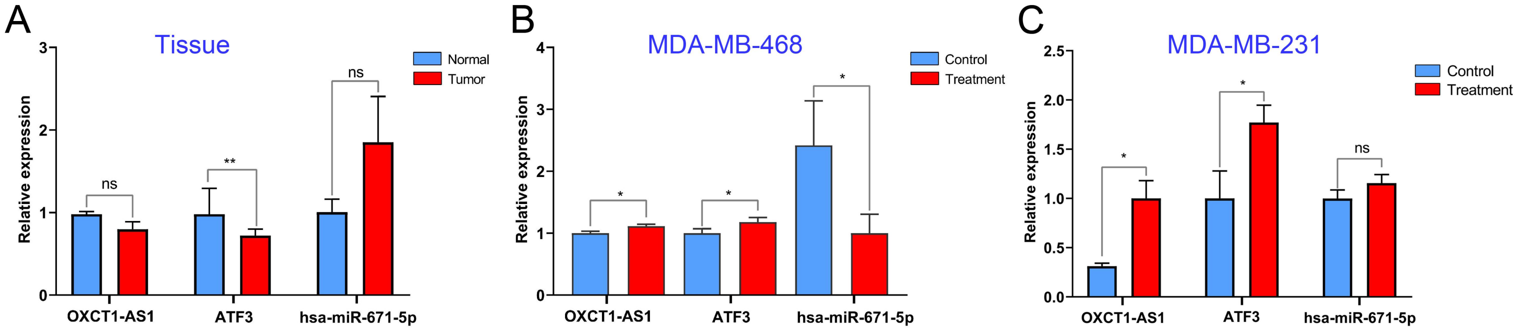

Figure S3 The RT-qPCR analysis was used to verify the expression levels of 6 genes, including LINC00460, SLC7A5, hsa-miR-143-3p, OXCT1-AS1, ATF3 and hsa-miR-671-5p, between tumor and normal, and between BEZ235-treated and control group, respectively (Note: “ *** ” : P<0.001, “ ** ” : P < 0.01, “ * ” : P < 0.05, “ ns ” : P > 0.05).

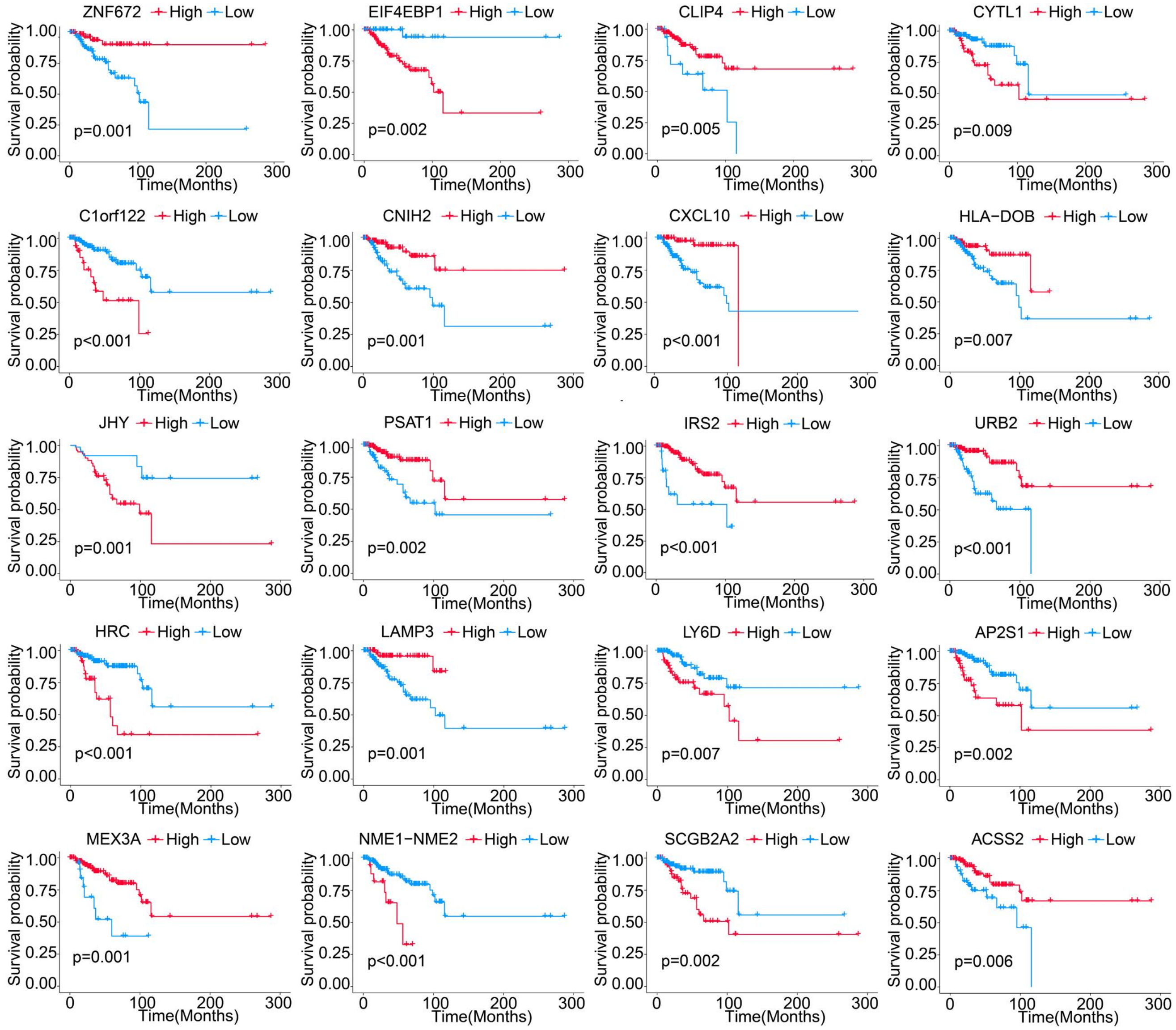

Figure S4 Kaplan-Meier curves showing overall survival of patients in the high- and low-expression groups of BEZ235 response-related DEmRNAs. Statistical significance tested using two-sided log-rank test. DEmRNAs: Differentially expressed mRNAs.

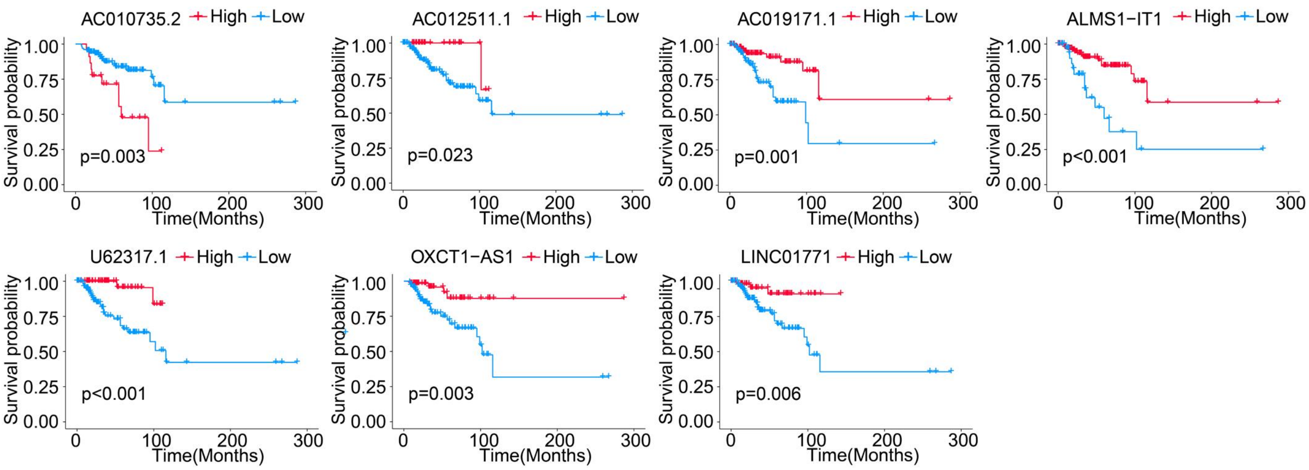

Figure S5 Kaplan-Meier curves showing overall survival of patients in the high- and low-expression groups of BEZ235 response-related DElncRNAs. Statistical significance tested using two-sided log-rank test. DElncRNAs: Differentially expressed lncRNAs.

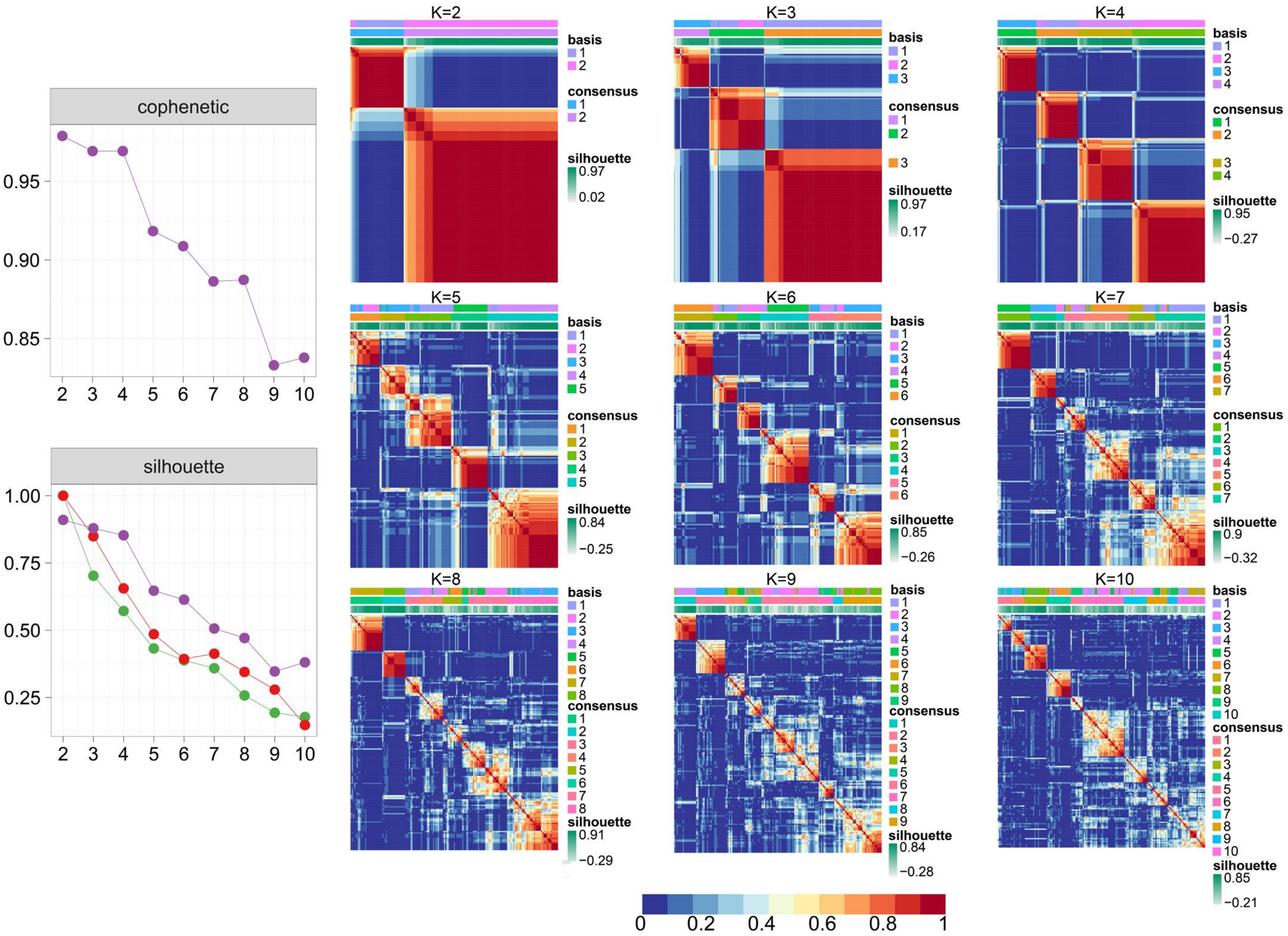

Figure S6, related to **Figure** 4. NMF rank survey of the TCGA-TNBC specimens using the 23 BEZ235 response-related genes. TCGA: The Cancer Genome Atlas, TNBC: triple negative breast cancer, NMF: non-negative matrix factorization.

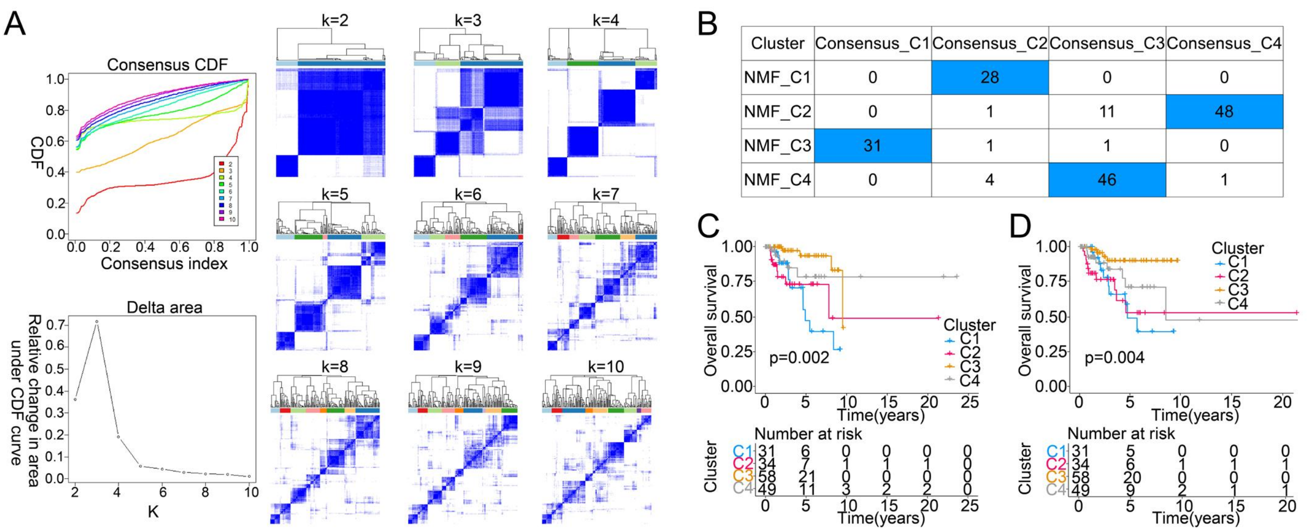

Figure S7 Consensus clustering analysis of the TCGA-TNBC specimens using the 23 BEZ235 response-related genes. **(A)** Consensus clustering matrix for k valued 2 to 10. **(B)** a 5x5 table summarized the number of TCGA-TNBC specimens that were assigned to each of the subgroups identified using NMF clustering or Consensus clustering, where the intersection (i.e., overlap) was marked in red. **(C, D)** Kaplan–Meier overall survival (OS) curve and progression-free survival (PFS) curve for 172 TCGA-TNBC patients of different clusters.

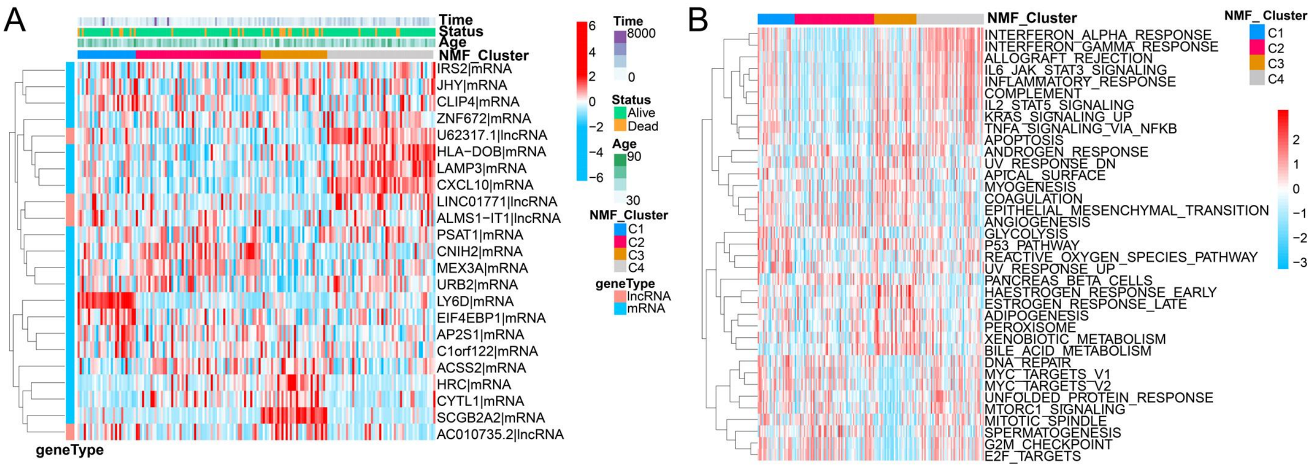

Figure S8 Characteristic analysis of four subtypes in the TCGA-TNBC cohort. **(A)** Heatmap of BEZ235 response-related genes expression between four clusters. **(B)** Heatmap of Hallmark pathways between four clusters.

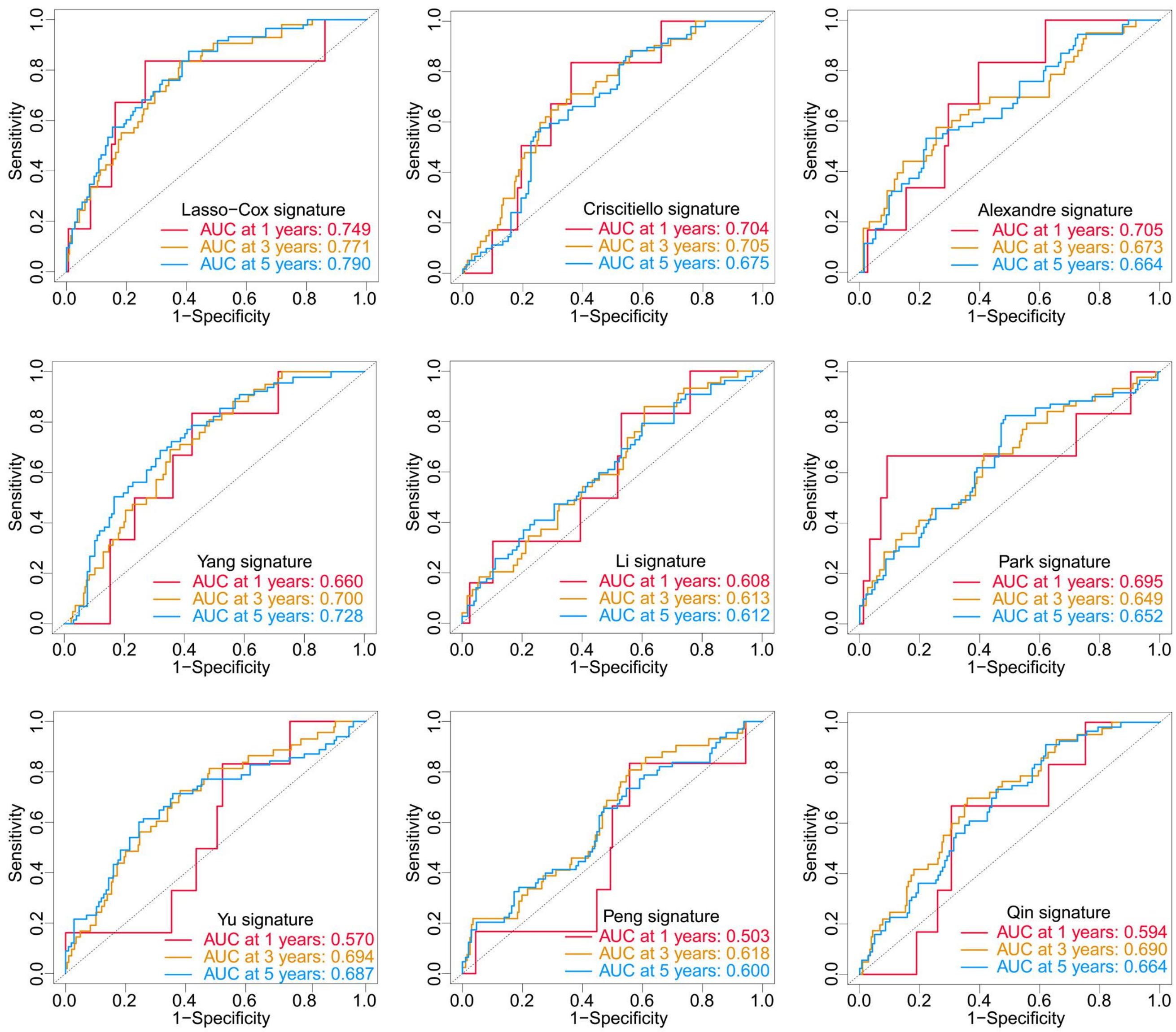

Figure S9 Time-dependent ROC analysis.

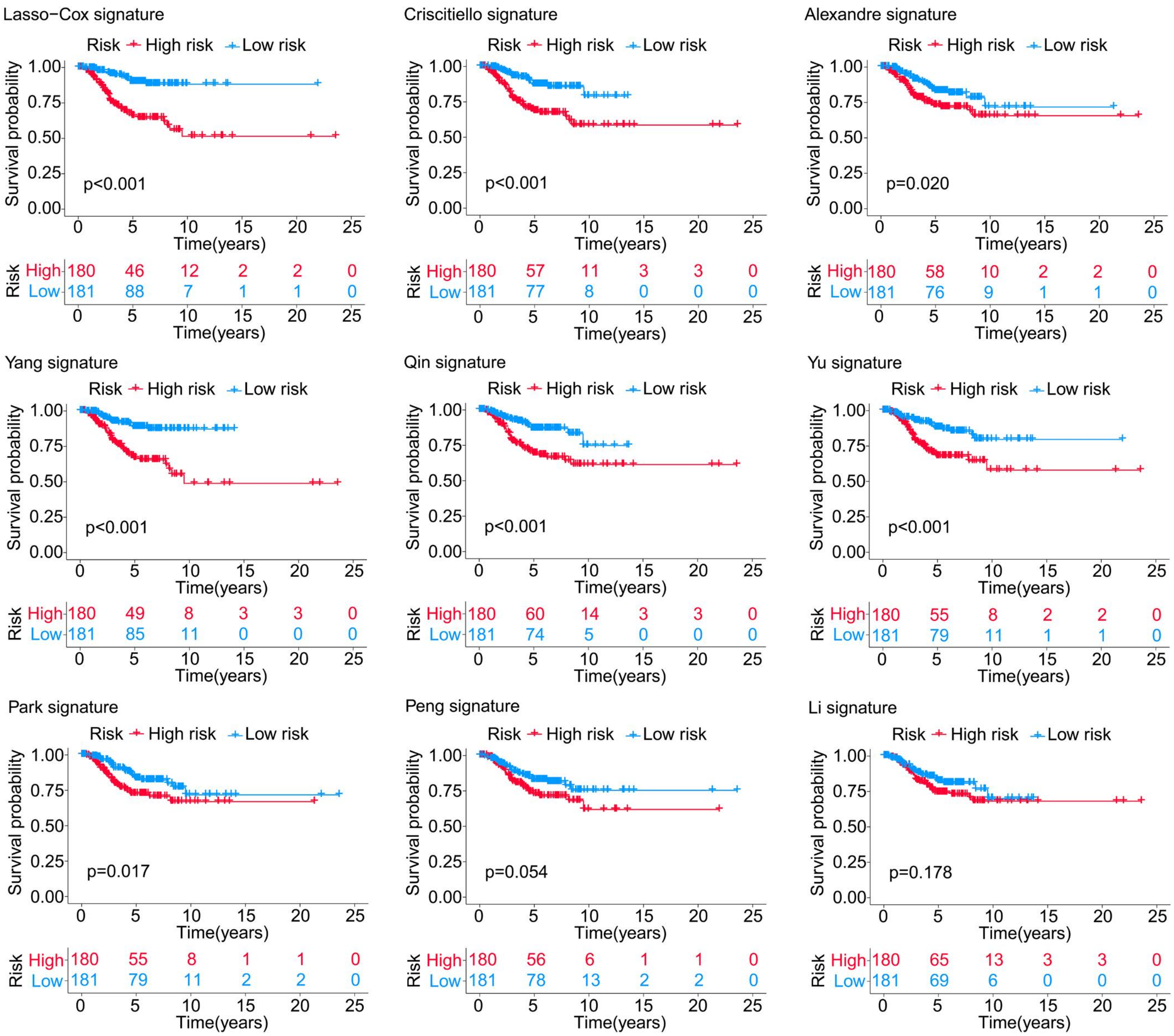

Figure S10 Kaplan–Meier survival analysis.

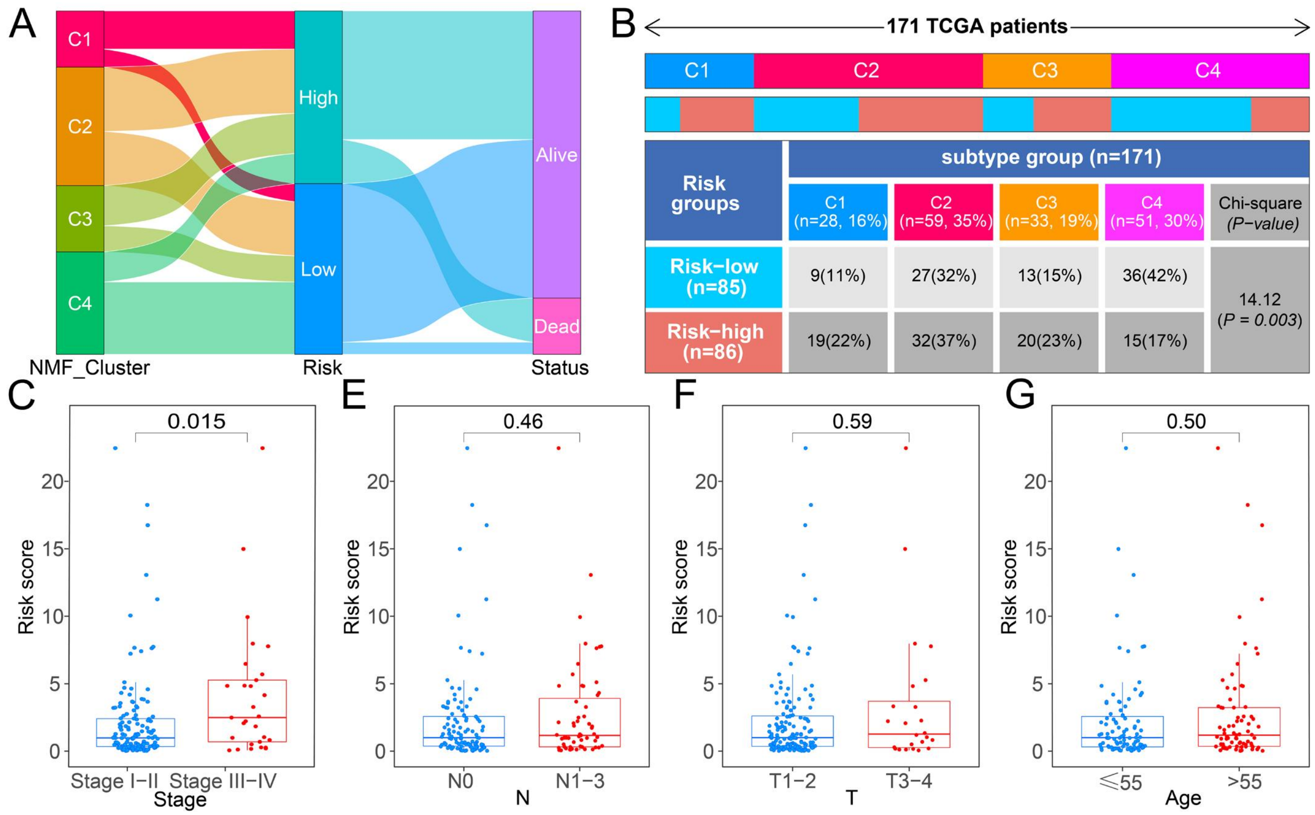

Figure S1 1 Correlation analysis between clinicopathological features and Risk score. **(A)** The Sankey plot indicated the association between NMF clustering, risk grouping, and survival status. **(B)** The Chis-quare test indicated that the Risk score was significantly different among the four molecular subgroups . **(C-F)** The difference of risk score in the clinical subgroup, including Stage I-II vs. Stage III-IV, N0 vs. N 1-3, T1-2 vs. T3-4, Age <= 55 vs. Age > 55.
