# Supplementary material for: Identification and panoramic analysis of drug response-related genes in triple negative breast cancer using as an example NVP-BEZ235
Source: Sci Rep. 2023 Apr 12;13:5984. doi: 10.1038/s41598-023-32757-4 (PMC10097725; doi:10.1038/s41598-023-32757-4)
Supplement: Supplementary file 2 — Supplementary Table S1. [file 41598_2023_32757_MOESM2_ESM.pdf]

Table S1 The gene list of 451 BEZ235 response-related genes associated with tumor characteristics.

Gene		Tumor vs. Normal		Treatment vs.Control	
		**log_2FC**	***P_{FDR}***	**log_2FC**	***P_{FDR}***
mRNAs (N = 365)	ABAT	-1.25	3.80E-25	1.21	1.01E-04
	ABCA1	-1.35	1.75E-22	2.04	8.13E-06
	ABCA5	-1.60	8.15E-37	2.62	1.72E-07
	ACAD11	-1.29	4.00E-26	1.31	1.37E-05
	ACKR4	-1.73	6.03E-23	1.42	5.87E-06
	ACSS2	-1.48	2.39E-31	1.11	5.43E-04
	ADAMTS1	-1.76	1.11E-30	2.01	6.97E-06
	ADAMTS4	1.29	1.03E-17	-2.62	3.35E-07
	ADHFE1	-1.92	7.96E-35	1.17	1.01E-04
	ALB	-5.15	1.25E-23	3.06	4.72E-07
	ALDH1A2	-2.88	3.37E-41	1.70	1.01E-05
	AMMECR1	1.11	3.34E-23	-1.32	9.61E-06
	AMOTL2	-1.34	5.09E-34	1.00	8.86E-05
	AMY2B	-1.41	1.20E-29	1.36	2.84E-05
	ANAPC11	1.26	1.69E-29	-1.10	1.75E-05
	ANG	-1.33	1.04E-28	1.30	1.99E-04
	ANKMY2	-1.06	1.94E-35	1.54	2.94E-05
	ANKRA2	-1.01	1.37E-30	1.11	1.20E-05
	ANKRD22	2.22	2.19E-21	-2.32	4.12E-07
	ANKRD31	-1.10	5.96E-20	3.09	9.38E-06
	AOX1	-2.89	5.40E-36	1.03	9.98E-04
	AP1S3	1.13	7.93E-11	-1.64	3.15E-06
	AP2S1	1.12	1.98E-31	-1.19	3.88E-05
	AR	-1.35	8.82E-25	1.01	3.72E-04
	ARHGEF28	-1.15	1.61E-29	1.04	8.95E-06
	ARID4A	-1.07	8.21E-31	1.34	1.68E-05
	ARID5B	-1.54	4.16E-37	1.10	1.03E-05
	ARMC6	1.13	1.62E-28	-1.38	1.88E-05
	ASNS	1.81	2.63E-32	-1.10	1.63E-05
	ASPHD2	1.04	9.55E-14	-1.65	6.18E-06
	ATF3	-2.02	6.66E-23	1.58	1.89E-06
	ATP1B3	1.07	6.05E-24	-1.14	9.45E-06
	BCL11B	1.07	2.77E-07	-1.10	1.06E-03
	BMP4	-1.52	1.73E-34	1.49	8.48E-06
	BOLA3	1.14	2.14E-32	-1.16	6.86E-06
	BTBD8	-1.08	7.09E-20	1.46	3.71E-06
	C1orf122	1.06	3.47E-24	-1.35	1.21E-05
	C1QL4	2.94	5.97E-12	-1.05	8.71E-04
	C4orf48	2.02	1.79E-23	-1.53	2.00E-05
	C5	-1.11	6.00E-25	1.88	1.96E-06
	C5orf46	2.89	9.48E-20	-1.60	3.59E-06
	CA9	6.26	4.07E-30	-1.85	1.70E-06
	CABCOCO1	-1.28	6.38E-22	1.83	1.64E-06
	CALCOCO1	-1.50	1.53E-41	1.49	6.15E-04

CALML5	3.63	3.29E-10	-1.26	2.81E-05
CALU	1.22	7.41E-28	-1.19	2.58E-03
CAMK2N2	2.87	8.95E-29	-1.11	6.41E-05
CAMP	4.08	1.36E-20	-3.26	1.13E-05
CAPN14	1.32	3.72E-03	-1.04	9.48E-05
CAPN15	1.13	5.40E-28	-1.01	1.24E-02
CAPN8	-2.08	5.25E-22	2.36	9.07E-07
CASP14	8.00	6.93E-11	-1.74	1.24E-06
CC2D2A	-1.08	5.33E-35	1.23	3.95E-05
CCDC124	1.08	7.38E-20	-1.19	2.17E-04
CCDC148	-1.01	4.18E-12	1.34	1.90E-05
CCDC167	2.32	5.30E-41	-1.40	6.44E-06
CCDC28B	1.39	2.16E-18	-1.47	1.16E-05
CCNI2	1.88	4.49E-04	-1.25	1.81E-04
CDC25A	3.14	1.45E-40	-1.11	1.84E-05
CDK2AP1	1.50	1.80E-30	-1.46	3.45E-06
CDKN1C	-1.55	6.84E-30	1.04	6.84E-05
CDNF	-1.36	3.26E-34	1.29	1.47E-05
CENPX	1.44	8.34E-29	-1.16	1.18E-05
CEP112	-1.66	6.37E-38	1.32	8.54E-06
CEP126	-2.43	3.41E-40	1.24	2.13E-04
CFAP43	-1.38	6.91E-17	1.63	6.89E-06
CFAP69	-2.01	2.26E-37	2.19	9.67E-07
CFAP70	-1.73	5.60E-31	1.92	4.23E-06
CFL1	1.18	2.90E-37	-1.72	1.02E-05
CHAC1	2.39	8.88E-33	-1.21	9.25E-06
CHAC2	1.58	9.01E-30	-1.85	3.90E-06
CHI3L2	2.20	6.72E-07	-1.45	2.87E-06
CHRDL2	2.89	1.67E-05	-1.62	1.74E-06
CHST4	2.07	1.58E-03	-1.30	2.38E-05
CIRBP	-1.16	2.26E-32	2.32	1.37E-06
CITED2	-1.25	1.07E-31	1.20	5.71E-05
CKAP4	1.22	1.61E-32	-1.02	5.99E-05
CKMT1B	1.92	3.85E-06	-1.11	2.48E-05
CLIC5	-2.42	2.26E-39	2.08	1.40E-05
CLIP4	-1.02	5.63E-24	1.04	1.61E-05
CLK1	-1.03	5.76E-27	1.16	9.71E-06
CLK4	-1.06	2.74E-29	1.38	4.04E-06
CMPK2	1.83	2.77E-16	-1.24	9.57E-05
CMYA5	-3.27	8.01E-28	1.75	1.16E-06
CNIH2	4.16	8.71E-39	-1.51	1.95E-05
COPE	1.17	1.62E-28	-1.02	5.46E-05
CORO1A	1.61	1.57E-18	-2.76	1.22E-06
COTL1	1.65	5.10E-25	-1.40	1.53E-05
CPO	-1.52	9.59E-27	2.39	1.52E-06
CRABP1	2.62	1.67E-03	-2.29	2.50E-06
CRABP2	1.75	1.27E-04	-2.18	2.44E-06
CREBRF	-1.50	5.23E-38	2.30	3.37E-07
CRELD2	1.24	1.81E-28	-1.00	1.62E-05

CSK	1.18	1.08E-33	-1.00	4.13E-04
CST6	2.74	3.87E-14	-1.10	3.53E-05
CTHRC1	2.36	1.67E-34	-1.12	1.37E-04
CXCL10	4.92	4.47E-35	-1.28	3.28E-05
CXCR3	2.08	2.45E-14	-1.00	3.48E-05
CYBA	1.51	1.64E-21	-1.89	1.52E-06
CYTL1	-1.39	3.28E-24	1.50	1.24E-05
DAB2	-1.12	8.68E-26	1.05	5.10E-05
DACH1	-2.48	8.45E-36	1.22	6.11E-05
DCPS	1.10	4.46E-29	-1.05	2.19E-04
DENND4C	-1.02	5.36E-26	1.06	4.65E-05
DGLUCY	-1.01	9.03E-33	1.16	1.23E-05
DHRS12	-1.02	2.09E-35	1.13	8.78E-06
DMGDH	-2.63	5.15E-33	2.70	5.08E-07
DNAAF9	-1.32	7.02E-38	1.25	3.43E-05
DNAH5	-1.45	7.91E-16	3.19	4.46E-07
DNAI7	-1.46	3.91E-21	1.02	3.19E-05
DNASE1L3	-2.48	3.44E-34	3.45	5.60E-07
DNPH1	1.57	8.40E-28	-1.74	8.47E-06
DPP4	-1.31	1.10E-23	2.89	4.39E-07
DPY19L2	-1.25	9.90E-27	1.41	1.12E-05
DRAP1	1.22	1.70E-23	-1.40	3.70E-05
DYNC2H1	-1.64	1.59E-33	1.53	2.97E-06
E2F2	3.52	4.97E-41	-1.70	1.25E-05
EDN2	3.14	4.47E-21	-3.30	1.19E-07
EFCAB6	-1.72	7.06E-30	3.14	1.29E-06
EFHB	-1.49	3.61E-16	1.76	4.35E-06
EGF	-1.86	6.49E-15	1.25	7.73E-06
EIF4B	-1.01	2.77E-36	1.04	3.20E-05
EIF4EBP1	2.11	4.93E-31	-1.15	7.55E-06
EIF5A	1.13	2.18E-34	-1.50	1.00E-05
ERO1A	1.07	6.71E-13	-1.18	4.86E-06
ETV1	-1.06	1.57E-18	1.25	5.12E-05
F11R	1.31	6.36E-39	-1.54	1.95E-05
F3	-1.66	1.76E-33	1.33	1.13E-05
FAM214A	-1.46	1.07E-32	1.32	5.08E-06
FILIP1	-1.32	1.12E-24	2.44	4.16E-06
FMO2	-2.18	1.44E-35	3.98	1.37E-07
FNIP2	-1.01	3.53E-25	1.04	2.24E-05
FREM1	-2.94	1.92E-39	1.46	1.93E-04
FRY	-1.79	2.09E-38	3.11	1.14E-06
FUT3	2.75	1.20E-18	-1.27	4.59E-04
GABARAPL1	-1.22	6.01E-35	1.82	9.22E-07
GALNT16	-1.69	6.83E-31	1.24	4.25E-04
GJB5	1.72	1.21E-02	-1.06	2.18E-04
GLIPR1L2	-1.84	6.83E-34	1.16	1.89E-04
GLRX2	1.23	2.22E-30	-1.21	3.57E-05
GNG5	1.03	3.07E-31	-1.12	2.72E-05
GPATCH4	1.01	2.21E-24	-1.06	4.36E-04

GPI	1.04	7.46E-23	-1.23	1.72E-05
GPR37	1.54	1.41E-15	-1.78	5.49E-05
GPRIN1	3.43	1.98E-41	-1.27	2.15E-05
GRAMD1C	-1.37	9.34E-16	1.35	9.46E-06
GRHL3	2.12	3.41E-15	-3.77	6.22E-07
HBQ1	2.70	1.12E-03	-1.45	1.47E-05
HES6	2.92	1.70E-34	-1.36	2.30E-03
HLA-DOB	1.73	1.82E-16	-1.17	7.24E-06
HMBS	1.10	5.28E-30	-1.01	9.16E-05
HNMT	-1.58	1.37E-39	1.56	2.43E-06
HPDL	3.51	8.74E-29	-1.49	2.54E-05
HPX	-1.25	6.24E-13	1.04	6.94E-05
HRC	-3.18	3.46E-38	1.62	8.95E-06
HYOU1	1.14	7.18E-25	-1.18	4.58E-06
IFI6	2.62	4.43E-27	-1.36	1.17E-04
IFITM1	1.06	5.79E-06	-1.42	8.34E-06
IGFBPL1	3.44	3.96E-16	-1.36	1.19E-05
IGSF23	4.14	4.69E-14	-1.03	2.51E-04
IL6	-2.29	1.89E-15	3.57	1.41E-06
INPP4B	-1.85	4.34E-28	1.70	9.67E-07
IQUB	-2.19	5.32E-29	1.87	1.62E-05
IRS2	-1.47	3.02E-29	2.04	1.84E-06
ITGA1	-1.44	5.51E-31	2.00	1.46E-06
JHY	-1.22	3.02E-32	1.24	1.35E-04
KCNK12	1.90	5.62E-12	-1.22	3.56E-04
KDELR3	1.48	8.95E-25	-1.67	9.38E-06
KISS1R	2.48	2.53E-07	-1.37	1.64E-02
KLF6	-1.09	1.31E-20	1.15	5.39E-05
KLHDC1	-2.13	1.33E-41	2.18	3.79E-07
KLHL31	-2.98	1.85E-31	1.22	3.64E-05
KRT16	3.88	2.43E-13	-1.14	1.33E-05
KRT83	2.76	2.30E-05	-1.75	3.41E-06
LAMP3	3.14	1.59E-30	-1.15	1.08E-05
LAPTM4B	1.68	5.98E-32	-1.41	1.84E-06
LEPR	-3.32	2.58E-41	1.12	2.44E-04
LETMD1	-1.15	1.59E-39	1.49	6.32E-06
LRP8	2.65	2.71E-37	-1.82	8.29E-07
LRRC10B	1.59	2.99E-02	-1.02	1.31E-04
LRRC17	-1.14	5.52E-26	2.95	1.72E-07
LRRC2	-3.24	1.68E-40	2.65	3.91E-07
LRRC59	1.24	1.66E-38	-1.41	1.85E-05
LY6D	3.47	6.05E-03	-1.79	1.84E-06
LY6E	1.73	2.52E-19	-1.64	2.75E-05
MAB21L4	3.15	8.96E-12	-1.09	8.01E-05
MAGEB4	3.34	7.29E-03	-1.06	5.46E-05
MAL2	1.55	4.40E-25	-1.06	1.90E-05
MANF	1.37	1.35E-37	-1.31	3.57E-06
MAOA	-4.03	3.84E-41	1.79	3.79E-05
MCM10	4.20	2.80E-41	-1.15	2.71E-05

ME3	-1.31	7.69E-35	1.20	3.61E-05
MESP2	2.11	9.48E-08	-2.18	1.22E-06
MEX3A	3.16	1.46E-30	-1.20	2.37E-04
MGARP	-2.29	1.25E-35	1.34	2.09E-05
MIF	1.69	9.77E-25	-1.13	3.61E-05
MINDY2	-1.07	6.23E-26	1.21	6.48E-05
MOXD1	1.89	6.16E-12	-1.15	1.68E-04
MROH8	-1.50	2.44E-39	1.70	7.57E-06
MRPL12	1.96	7.91E-30	-1.62	5.29E-06
MRPL4	1.09	5.34E-25	-1.07	2.91E-05
MTMR10	-1.47	2.19E-39	2.24	5.08E-07
MUC1	1.21	8.44E-05	-1.99	5.60E-07
MUC5B	2.98	1.62E-16	-1.23	5.98E-05
MYBL2	4.80	4.67E-42	-1.39	6.38E-06
MYBPC1	-2.65	4.21E-10	1.78	2.42E-06
MYH3	-1.26	7.73E-25	1.51	5.58E-06
MYO5C	-1.01	1.31E-21	1.92	5.99E-07
MYOM1	-4.07	2.87E-41	2.23	1.23E-06
N4BP2L1	-1.41	2.60E-33	2.24	1.42E-06
NATD1	-1.81	4.27E-39	2.30	5.08E-07
NCS1	1.47	3.04E-30	-1.25	1.07E-04
NDUFA9	1.01	6.32E-27	-1.02	1.61E-05
NDUFS6	1.20	5.31E-23	-1.33	7.23E-06
NEDD9	-1.38	1.19E-27	1.46	4.19E-06
NEK10	-2.92	6.27E-25	2.78	6.02E-07
NEK9	-1.08	1.19E-40	1.01	2.90E-05
NEXN	-1.53	3.38E-31	2.67	4.10E-07
NIPSNAP3B	-2.33	2.16E-38	1.08	1.15E-05
NME1	1.93	4.52E-40	-1.65	4.09E-06
NME1-NME2	1.08	3.08E-12	-2.02	2.98E-06
NPHP3	-1.09	1.35E-29	1.22	4.18E-06
NRXN1	-1.32	1.77E-24	1.58	5.49E-06
NT5DC2	1.54	1.99E-29	-1.45	1.73E-05
NTHL1	1.09	1.90E-17	-1.08	2.08E-03
NUDT1	1.56	1.44E-33	-1.29	4.10E-06
ONECUT2	3.92	2.35E-21	-1.18	1.51E-04
OPRK1	3.58	4.09E-10	-1.69	2.43E-06
OVCH2	-3.04	4.62E-22	1.82	1.63E-06
PACSIN1	2.54	5.50E-29	-1.85	1.59E-04
PADI2	1.12	1.72E-08	-1.57	5.06E-06
PAMR1	-3.88	4.67E-42	2.61	1.18E-06
PCDH19	-2.09	5.05E-38	1.88	7.33E-06
PDAP1	1.02	1.45E-32	-1.05	1.98E-05
PDE1C	-1.61	1.08E-22	1.51	1.93E-06
PDE5A	-1.71	4.60E-34	1.68	1.26E-06
PDK3	1.30	2.06E-10	-1.06	1.91E-05
PDK4	-4.25	3.18E-41	2.91	5.37E-07
PDLIM4	-1.05	8.16E-15	1.10	4.39E-03
PDXP	1.39	7.73E-16	-1.02	1.68E-04

PDZK1	-2.05	5.88E-21	2.03	7.19E-06
PDZK1IP1	1.66	6.19E-07	-1.05	1.22E-04
PFN2	1.47	8.95E-21	-1.37	3.89E-06
PHLDA2	1.85	3.69E-19	-2.29	1.38E-06
PIK3IP1	-1.08	8.60E-29	2.85	1.28E-06
PIP	-1.64	6.42E-19	1.45	6.59E-06
PITX1	3.84	1.19E-35	-1.42	8.17E-05
PKM	1.17	3.75E-30	-1.49	2.95E-05
PLA2R1	-1.80	1.05E-33	2.62	4.77E-07
PLAC1	3.60	3.34E-22	-3.62	4.02E-07
PLCH1	1.72	2.05E-17	-2.18	2.74E-05
PLEKHH2	-1.73	1.26E-33	1.46	2.44E-04
PLEKHN1	1.50	2.02E-14	-1.19	1.40E-04
PLIN1	-6.21	2.33E-39	1.19	2.45E-05
PNP	1.55	1.49E-34	-1.33	5.68E-06
POLR3K	1.14	1.33E-30	-1.10	1.37E-05
PPIL1	1.08	5.66E-28	-1.21	1.07E-05
PPP1R15A	-1.03	2.43E-20	1.50	3.17E-06
PRR19	2.08	2.03E-37	-1.70	4.32E-06
PRSS1	2.44	1.37E-08	-1.87	4.86E-06
PRX	-1.52	6.50E-30	1.17	1.19E-04
PSAT1	2.53	7.73E-27	-1.17	1.43E-05
PSMB3	1.05	8.34E-29	-1.23	5.08E-06
PSMC4	1.24	6.46E-39	-1.27	5.01E-06
PTPN21	-1.42	2.99E-38	1.25	3.95E-05
PUSL1	1.59	4.00E-33	-1.15	4.19E-05
PYCR1	2.46	2.33E-38	-1.42	8.83E-05
PYROXD2	-1.14	1.05E-23	1.32	2.23E-05
RAB5IF	1.42	5.87E-39	-1.10	8.09E-06
RANBP1	1.44	7.32E-39	-1.06	1.29E-05
RASSF6	-1.92	4.14E-18	1.46	7.56E-06
RASSF9	-1.55	1.36E-28	1.49	3.51E-06
RELN	-1.67	1.23E-34	5.21	1.19E-07
RETSAT	-1.97	3.12E-29	1.07	2.04E-05
RGCC	-1.08	4.68E-26	2.41	4.59E-07
RGS20	2.13	4.35E-07	-1.21	1.68E-04
RGS5	-1.56	7.96E-35	1.36	2.17E-05
RHOBTB3	-1.14	2.31E-18	1.10	1.36E-05
RHOXF1	-2.65	2.65E-33	1.24	6.36E-05
RNASE4	-2.11	4.86E-36	1.33	8.44E-06
RPP21	1.22	2.26E-28	-1.09	1.13E-05
RRBP1	1.01	7.36E-23	-1.08	8.10E-06
RUNDC3B	-2.19	6.46E-39	1.24	2.15E-05
RUNX2	1.10	4.76E-10	-1.51	2.05E-02
S100A16	1.20	8.36E-09	-1.15	1.15E-04
S100A7	3.41	1.89E-09	-1.30	9.73E-06
S100A7A	4.73	2.19E-09	-1.56	8.04E-06
S100A8	4.96	8.98E-11	-3.41	1.37E-07
S100A9	3.89	1.48E-10	-2.14	1.10E-06

Table Continued

SAA2-SAA4	-2.72	1.44E-24	2.78	1.72E-07
SAMD4A	-1.16	1.22E-22	1.94	1.97E-06
SCGB2A2	-1.22	1.98E-23	2.05	1.90E-06
SDF2L1	1.60	6.01E-30	-1.61	3.41E-06
SECTM1	2.09	6.20E-15	-1.54	6.94E-04
SERPINE2	1.98	5.03E-17	-1.18	2.74E-05
SH3BGRL3	1.03	4.14E-25	-1.18	1.09E-04
SLC10A4	2.39	8.96E-08	-1.21	6.08E-03
SLC16A1	1.31	3.77E-09	-1.94	2.44E-06
SLC16A10	1.50	2.50E-15	-1.30	3.90E-06
SLC1A7	-2.43	5.71E-32	3.21	3.37E-07
SLC27A1	-1.15	1.34E-34	1.12	8.15E-06
SLC28A1	1.96	1.36E-03	-1.07	4.27E-05
SLC39A7	1.17	4.70E-31	-1.02	5.60E-04
SLC7A11	2.39	8.94E-26	-2.18	9.67E-07
SLC7A5	3.72	1.03E-41	-1.67	2.99E-06
SLFNL1	1.54	2.84E-12	-1.08	9.57E-04
SLURP1	4.22	3.00E-15	-1.56	1.30E-05
SNRPD1	1.31	3.02E-36	-1.27	4.83E-06
SOX11	4.45	1.78E-28	-1.17	6.94E-05
SPATA9	-1.81	3.13E-26	2.16	4.30E-06
SPEF2	-1.15	1.05E-20	2.66	2.81E-05
SRM	1.02	2.45E-22	-1.30	1.44E-05
SRPX2	-1.00	5.27E-10	1.15	2.35E-05
SRSF12	1.87	6.58E-13	-1.91	6.57E-07
STARD13	-1.25	8.40E-34	1.38	1.82E-06
SUSD4	1.45	9.26E-08	-1.29	1.83E-04
SYBU	-1.86	1.54E-34	1.16	3.01E-05
TAP2	1.42	2.72E-17	-1.02	1.68E-05
TBX15	-2.19	2.60E-33	1.23	4.35E-05
TCP11L2	-1.64	5.01E-34	1.30	4.92E-05
THSD7A	-1.35	3.29E-27	1.62	1.48E-05
TIMM17B	1.17	2.66E-30	-1.18	8.52E-06
TIMP1	1.12	1.15E-14	-1.18	9.17E-06
TMED9	1.02	1.63E-27	-1.17	8.25E-05
TMEM135	-1.21	1.82E-31	1.66	3.00E-06
TMEM171	3.63	1.88E-31	-1.42	3.92E-04
TMEM232	-1.98	1.39E-34	2.08	1.20E-05
TMEM270	4.42	8.94E-28	-1.35	3.61E-05
TMEM40	1.87	2.98E-05	-1.10	2.29E-05
TMSB15A	3.51	8.41E-22	-1.86	6.65E-06
TOMM5	1.05	1.39E-30	-1.31	5.29E-06
TOR3A	1.15	1.25E-35	-1.03	4.83E-05
TPBGL	1.79	2.96E-19	-1.38	3.83E-06
TRIM63	-3.22	2.68E-18	1.87	1.17E-05
TRIM66	-1.27	2.92E-31	2.04	2.78E-06
TRPC1	-1.28	4.77E-31	2.36	1.88E-06
TSEN15	1.02	7.50E-33	-1.43	2.98E-06
TSPO	1.02	5.97E-17	-1.33	4.37E-05

	TTC23L	-1.05	7.90E-10	2.48	1.29E-05
	TTC6	-2.13	5.05E-19	1.17	3.55E-05
	TUBA4A	1.29	5.92E-15	-1.05	2.99E-05
	TUBG1	1.22	1.12E-30	-1.06	7.67E-06
	UCHL1	2.03	3.57E-06	-1.11	8.44E-06
	UHRF1	3.74	7.25E-42	-1.48	4.80E-06
	UQCC2	1.28	1.50E-32	-1.80	2.73E-06
	UQCC3	1.35	3.20E-23	-1.09	3.77E-04
	URB2	1.06	1.87E-22	-1.34	1.08E-05
	USH1G	2.58	4.73E-07	-1.25	1.51E-04
	USHBP1	-2.28	5.17E-40	1.54	1.38E-05
	USP44	-1.67	1.87E-28	2.18	2.11E-06
	UST	-1.01	1.41E-25	1.26	9.21E-06
	VOPP1	1.16	1.54E-17	-1.03	1.33E-05
	VPS13C	-1.06	1.25E-26	1.43	4.95E-05
	VWCE	-1.34	2.58E-29	1.29	2.01E-05
	WDR19	-1.33	3.23E-37	2.24	1.32E-06
	WDR4	1.36	3.60E-33	-1.14	8.82E-06
	WNK4	-1.33	6.15E-14	1.34	1.76E-05
	YPEL2	-1.00	3.13E-26	1.77	1.42E-06
	ZCWPW2	-1.04	3.22E-31	1.73	4.75E-05
	ZDHHC17	-1.09	7.72E-32	1.23	3.20E-05
	ZDHHC2	-1.09	8.20E-25	2.10	5.97E-06
	ZFP2	-1.32	1.88E-33	2.81	1.21E-05
	ZHX1-C8orf76	1.61	1.31E-27	-1.02	3.95E-05
	ZNF672	1.06	3.63E-30	-1.01	1.32E-05
	ZSCAN23	-1.33	4.40E-19	1.79	4.90E-06
lncRNAs (N = 66)	AC002451.1	-2.56	8.40E-37	1.62	2.14E-05
	AC004540.1	-1.09	2.82E-23	1.33	3.22E-04
	AC005062.1	-1.92	4.36E-12	1.83	1.03E-04
	AC005740.4	-1.28	1.49E-31	1.42	6.60E-05
	AC006273.1	-1.12	1.30E-17	1.45	4.99E-05
	AC010735.2	-1.50	3.61E-25	3.47	7.20E-07
	AC010976.2	-1.84	7.18E-25	2.62	2.08E-05
	AC010980.2	1.29	8.77E-03	-2.52	4.93E-06
	AC011479.2	1.95	1.84E-07	-1.02	6.87E-04
	AC012467.1	-1.39	1.21E-10	3.47	2.03E-06
	AC012511.1	-1.08	1.19E-16	1.71	2.24E-04
	AC019171.1	2.42	6.03E-04	-1.33	4.37E-05
	AC025034.1	-1.16	3.06E-20	1.66	1.39E-05
	AC044810.2	-2.36	1.19E-18	1.72	4.97E-05
	AC044849.1	-1.02	6.08E-06	1.01	5.93E-04
	AC046168.1	3.08	5.01E-08	-1.94	1.35E-05
	AC068580.2	3.64	1.04E-09	-1.78	2.44E-05
	AC092803.1	1.24	2.73E-16	-1.24	9.58E-04
	AC095055.1	-1.36	1.44E-31	1.37	1.75E-03
	AC113139.1	1.32	3.84E-12	-1.05	8.03E-04
	AC116312.1	-1.89	1.99E-20	1.45	7.15E-05
	AL031985.3	1.56	1.26E-31	-1.02	1.02E-04

	AL121672.3	1.29	5.62E-05	-1.46	2.08E-04
	AL139260.1	-1.25	2.49E-09	2.23	5.62E-06
	AL161457.2	-1.20	1.46E-27	1.72	1.97E-05
	AL356599.1	-1.15	2.17E-31	1.06	9.51E-05
	AL357992.1	2.18	1.27E-12	-1.00	3.65E-03
	AL359220.1	-1.30	2.36E-28	1.63	3.89E-05
	AL359715.2	-1.28	7.59E-23	1.66	3.17E-05
	AL390208.1	-1.60	2.07E-27	1.69	2.29E-04
	AL445524.1	2.02	7.04E-32	-1.02	2.17E-04
	ALMS1-IT1	1.22	3.81E-12	-1.11	1.83E-04
	AP000866.1	-1.58	7.79E-30	1.24	1.46E-03
	AP002761.3	-1.28	1.67E-28	1.56	4.08E-04
	AP002840.1	-1.56	2.49E-18	2.44	3.62E-06
	AP003071.1	-1.01	7.41E-11	1.16	5.18E-04
	C2-AS1	1.98	8.75E-10	-1.09	6.13E-05
	DGUOK-AS1	1.16	4.80E-11	-1.84	1.07E-05
	DSCR9	1.91	2.62E-19	-1.20	5.57E-04
	EIPR1-IT1	2.92	6.52E-22	-1.43	5.23E-05
	EPB41L4A-AS1	-1.27	9.28E-28	2.00	1.73E-05
	GAS1RR	-1.93	1.24E-32	1.62	4.43E-04
	KLF3-AS1	-1.66	1.98E-31	1.68	2.48E-05
	LINC00239	1.16	4.54E-12	-1.47	6.43E-05
	LINC00460	4.37	1.30E-22	-1.41	1.07E-03
	LINC00702	-2.08	4.66E-27	1.80	2.67E-05
	LINC00886	-1.32	6.58E-27	1.57	3.52E-05
	LINC00921	-1.05	8.58E-28	1.15	5.48E-05
	LINC01063	1.68	2.47E-18	-1.10	8.48E-05
	LINC01213	2.18	5.65E-06	-2.22	6.53E-06
	LINC01402	-2.18	1.11E-29	2.14	1.73E-05
	LINC01485	-3.44	1.72E-28	2.84	1.37E-05
	LINC01549	2.15	1.60E-04	-1.62	1.32E-04
	LINC01768	-2.27	9.60E-21	2.60	7.41E-06
	LINC01771	2.06	8.30E-13	-1.22	1.41E-04
	LINC01836	-1.04	1.66E-14	1.09	9.11E-05
	LINC01908	-1.91	1.60E-15	1.48	3.26E-05
	LINC02303	-1.43	5.00E-13	1.33	5.30E-05
	LINC02352	-1.35	2.76E-17	2.30	1.68E-04
	NDUFB2-AS1	1.14	1.40E-27	-1.76	3.45E-05
	NR2F1-AS1	-1.04	2.59E-23	1.05	2.24E-04
	OXCT1-AS1	-1.52	3.01E-29	1.58	1.36E-05
	PKP4-AS1	1.82	1.52E-07	-1.34	5.30E-05
	SFTA1P	-1.15	2.68E-09	1.60	1.40E-05
	U62317.1	4.20	2.67E-35	-1.30	5.10E-05
	Z97989.1	-1.54	6.58E-32	1.50	1.91E-05
miRNAs (N = 20)	hsa-miR-1277-3p	2.02	1.81E-29	-1.04	3.35E-02
	hsa-miR-136-3p	-1.12	8.40E-14	1.77	3.47E-02

Table Continued

hsa-miR-143-3p	-1.37	2.31E-22	1.74	3.91E-04
hsa-miR-1468-5p	-0.59	6.36E-05	2.10	3.93E-02
hsa-miR-148a-5p	0.74	1.82E-08	-0.79	1.23E-07
hsa-miR-15a-5p	0.50	6.28E-08	-0.52	2.63E-03
hsa-miR-17-3p	1.55	3.10E-29	-0.51	1.57E-02
hsa-miR-188-5p	2.30	4.18E-39	-1.02	1.81E-05
hsa-miR-18a-5p	2.54	3.27E-56	-0.58	4.19E-05
hsa-miR-19a-3p	1.99	3.84E-40	-0.82	1.05E-08
hsa-miR-218-5p	-1.33	2.53E-36	1.97	1.98E-05
hsa-miR-301a-5p	2.52	8.23E-42	-0.74	1.74E-03
hsa-miR-31-3p	0.81	2.14E-04	-0.67	6.30E-03
hsa-miR-323a-3p	-0.83	9.09E-06	2.58	3.98E-02
hsa-miR-33a-5p	2.14	1.56E-36	-0.61	2.23E-02
hsa-miR-379-5p	-1.83	1.30E-41	2.02	1.61E-02
hsa-miR-500a-5p	0.97	2.64E-13	-0.75	2.30E-03
hsa-miR-671-5p	1.86	9.89E-46	-0.75	4.01E-05
hsa-miR-769-3p	1.04	1.18E-14	-0.58	2.00E-02
hsa-miR-98-3p	1.53	3.98E-27	-0.84	2.05E-04
