# Supplementary material for: Identification and panoramic analysis of drug response-related genes in triple negative breast cancer using as an example NVP-BEZ235
Source: Sci Rep. 2023 Apr 12;13:5984. doi: 10.1038/s41598-023-32757-4 (PMC10097725; doi:10.1038/s41598-023-32757-4)
Supplement: Supplementary file 3 — Supplementary Table S2. [file 41598_2023_32757_MOESM3_ESM.pdf]

Table S2 The 46 differential pathways-associated and BEZ235 response-related mRNAs.

Gene symbol	Differential pathways involved
ATF3	HALLMARK_UV_RESPONSE_UP
CDKN1C	HALLMARK_UV_RESPONSE_UP
PDK4	HALLMARK_OXIDATIVE_PHOSPHORYLATION
CITED2	HALLMARK_GLYCOLYSIS
RETSAT	HALLMARK_OXIDATIVE_PHOSPHORYLATION
PPP1R15A	HALLMARK_MTORC1_SIGNALING
IL6	HALLMARK_UV_RESPONSE_UP
IRS2	HALLMARK_GLYCOLYSIS
ANG	HALLMARK_GLYCOLYSIS
MAOA	HALLMARK_UV_RESPONSE_UP
HMBS	HALLMARK_MTORC1_SIGNALING
GPI	HALLMARK_MTORC1_SIGNALING; HALLMARK_OXIDATIVE_PHOSPHORYLATION
SLC7A5	HALLMARK_MTORC1_SIGNALING
CHST4	HALLMARK_GLYCOLYSIS
PSMB3	HALLMARK_MYC_TARGETS_V1
AP2S1	HALLMARK_UV_RESPONSE_UP
PSMC4	HALLMARK_MTORC1_SIGNALING
ERO1A	HALLMARK_GLYCOLYSIS; HALLMARK_MTORC1_SIGNALING
LAMP3	HALLMARK_INTERFERON_ALPHA_RESPONSE
CFL1	HALLMARK_PI3K_AKT_MTOR_SIGNALING
CDC25A	HALLMARK_MTORC1_SIGNALING
TUBA4A	HALLMARK_UV_RESPONSE_UP; HALLMARK_MTORC1_SIGNALING
LY6E	HALLMARK_INTERFERON_ALPHA_RESPONSE
CORO1A	HALLMARK_MTORC1_SIGNALING
SDF2L1	HALLMARK_MTORC1_SIGNALING
ASNS	HALLMARK_UV_RESPONSE_UP; HALLMARK_MTORC1_SIGNALING
CXCL10	HALLMARK_INTERFERON_ALPHA_RESPONSE
PKM	HALLMARK_GLYCOLYSIS
SRM	HALLMARK_MYC_TARGETS_V2; HALLMARK_MYC_TARGETS_V1
NDUFA9	HALLMARK_OXIDATIVE_PHOSPHORYLATION
PSAT1	HALLMARK_MTORC1_SIGNALING
POLR3K	HALLMARK_GLYCOLYSIS
IFITM1	HALLMARK_INTERFERON_ALPHA_RESPONSE
PNP	HALLMARK_MTORC1_SIGNALING
SNRPD1	HALLMARK_MYC_TARGETS_V1
PDK3	HALLMARK_GLYCOLYSIS
MIF	HALLMARK_GLYCOLYSIS
KDELR3	HALLMARK_GLYCOLYSIS
SLC7A11	HALLMARK_MTORC1_SIGNALING
TUBG1	HALLMARK_MTORC1_SIGNALING
NME1	HALLMARK_MYC_TARGETS_V1
RANBP1	HALLMARK_MYC_TARGETS_V1
CMPK2	HALLMARK_INTERFERON_ALPHA_RESPONSE
GLRX2	HALLMARK_REACTIVE_OXYGEN_SPECIES_PATHWAY
NDUFS6	HALLMARK_OXIDATIVE_PHOSPHORYLATION
PDAP1	HALLMARK_UV_RESPONSE_UP; HALLMARK_MTORC1_SIGNALING
