# Supplementary material for: Identification and panoramic analysis of drug response-related genes in triple negative breast cancer using as an example NVP-BEZ235
Source: Sci Rep. 2023 Apr 12;13:5984. doi: 10.1038/s41598-023-32757-4 (PMC10097725; doi:10.1038/s41598-023-32757-4)
Supplement: Supplementary file 4 — Supplementary Table S3. [file 41598_2023_32757_MOESM4_ESM.pdf]

**Table S3** Targeting relationships in BEZ235-related ceRNA network

| miRNA | target | miRNA | target |
|---|---|---|---|
| hsa-miR-33a-5p | EPB41L4A-AS1 | hsa-miR-143-3p | CDC25A |
| hsa-miR-18a-5p | EPB41L4A-AS1 | hsa-miR-143-3p | GPI |
| hsa-miR-19a-3p | KLF3-AS1 | hsa-miR-143-3p | PNP |
| hsa-miR-15a-5p | SFTA1P | hsa-miR-143-3p | SRM |
| hsa-miR-19a-3p | EPB41L4A-AS1 | hsa-miR-143-3p | TUBG1 |
| hsa-miR-33a-5p | NR2F1-AS1 | hsa-miR-18a-5p | MAOA |
| hsa-miR-15a-5p | KLF3-AS1 | hsa-miR-19a-3p | RETSAT |
| hsa-miR-15a-5p | NR2F1-AS1 | hsa-miR-17-3p | RETSAT |
| hsa-miR-143-3p | LINC00460 | hsa-miR-143-3p | PSMC4 |
| hsa-miR-143-3p | EIPR1-IT1 | hsa-miR-500a-5p | ATF3 |
| hsa-miR-15a-5p | OXCT1-AS1 | hsa-miR-769-3p | ATF3 |
| hsa-miR-143-3p | NDUFB2-AS1 | hsa-miR-671-5p | ATF3 |
| hsa-miR-18a-5p | NR2F1-AS1 | hsa-miR-671-5p | PDK4 |
| hsa-miR-33a-5p | LINC00886 | hsa-miR-500a-5p | MAOA |
| hsa-miR-33a-5p | KLF3-AS1 | hsa-miR-671-5p | RETSAT |
| hsa-miR-33a-5p | GAS1RR | hsa-miR-33a-5p | IRS2 |
| hsa-miR-671-5p | GAS1RR | hsa-miR-143-3p | SLC7A11 |
| hsa-miR-769-3p | OXCT1-AS1 | hsa-miR-143-3p | HMBS |
| hsa-miR-671-5p | NR2F1-AS1 | hsa-miR-33a-5p | MAOA |
| hsa-miR-18a-5p | KLF3-AS1 | hsa-miR-19a-3p | CITED2 |
| hsa-miR-671-5p | LINC01485 | hsa-miR-17-3p | CITED2 |
| hsa-miR-769-3p | LINC01836 | hsa-miR-19a-3p | IRS2 |
| hsa-miR-188-5p | EPB41L4A-AS1 | hsa-miR-15a-5p | PDK4 |
| hsa-miR-769-3p | KLF3-AS1 | hsa-miR-17-3p | IRS2 |
| hsa-miR-769-3p | SFTA1P | hsa-miR-143-3p | SLC7A5 |
| hsa-miR-671-5p | OXCT1-AS1 | hsa-miR-188-5p | ATF3 |
| hsa-miR-671-5p | LINC00886 | hsa-miR-143-3p | PDAP1 |
| hsa-miR-500a-5p | EPB41L4A-AS1 | | |
| hsa-miR-769-3p | LINC00886 | | |
| hsa-miR-671-5p | KLF3-AS1 | | |
| hsa-miR-769-3p | LINC01485 | | |
| hsa-miR-769-3p | NR2F1-AS1 | | |
| hsa-miR-17-3p | NR2F1-AS1 | | |
| hsa-miR-671-5p | LINC02352 | | |
| hsa-miR-769-3p | LINC02352 | | |
| hsa-miR-15a-5p | AP003071.1 | | |
| hsa-miR-18a-5p | AC095055.1 | | |
| hsa-miR-15a-5p | EPB41L4A-AS1 | | |
| hsa-miR-15a-5p | IRS2 | | |
| hsa-miR-671-5p | IRS2 | | |
