# Supplementary material for: Identification and panoramic analysis of drug response-related genes in triple negative breast cancer using as an example NVP-BEZ235
Source: Sci Rep. 2023 Apr 12;13:5984. doi: 10.1038/s41598-023-32757-4 (PMC10097725; doi:10.1038/s41598-023-32757-4)
Supplement: Supplementary file 5 — Supplementary Table S4. [file 41598_2023_32757_MOESM5_ESM.pdf]

**Table S4** The primer sequences of RT-qPCR.

| Names | Sequences |
|---|---|
| LINC00460 | Forward: 5′-GGCATTGTAGAAAGACTGAGCG-3′ |
| | Reverse: 5′-TAGCATACGAATTTGGGTGGG-3′ |
| SLC7A5 | Forward: 5′-CCGTGAACTGCTACAGCGT-3′ |
| | Reverse: 5′-CTTCCCGATCTGGACGAAGC-3′ |
| ATF3 | Forward: 5′-CCTCTGCGCTGGAATCAGTC-3′ |
| | Reverse: 5′-TTCTTTCTCGTCGCCTCTTTTT-3′ |
| OXCT1-AS1 | Forward: 5′-CCTGGACTGCGTTCACGTTT-3′ |
| | Reverse: 5′-CTGCAAGCCTTGTTGCTCAC-3′ |
| hsa-miR-671-5p | Forward: 5′-AGGAAGCCCTGGAGGGGC-3′ |
| hsa-miR-143-3p | Forward: 5′-TGAGATGAAGCACTGTAGCTC-3′ |
| GAPDH | Forward: 5′-GAACGGGAAGCTCACTGG-3′ |
| | Reverse: 5′-GCCTGCTTCACCACCTTCT-3′ |

Note: Upstream and downstream primers of internal reference U6 and reverse primers of miRNA were delivered with TB Green™ Premix Ex Taq™ II.
